# The effect of dietary nitrate supplementation on resistance exercise performance: A dose–response investigation

**DOI:** 10.1007/s00421-025-05779-1

**Published:** 2025-04-24

**Authors:** Rachel Tan, Isabella G. Lincoln, Keonabelle K. Paniagua, Justin M. Foster, Lauren E. Wideen, Raymond T. Gerardo, Nathan J. Ornelas, Isaac Tchaprazian, Jeffrey Li, Michael Egiazarian, Samantha N. Rowland, Stephen J. Bailey, Adam Pennell

**Affiliations:** 1 Department of Sports Medicine, Natural Sciences Division, Pepperdine University, 24255 Pacific Coast Highway, Malibu, CA 90263 USA; 2 School of Sport, Exercise and Health Sciences, Loughborough University, Loughborough, UK

**Keywords:** Nitric oxide, Power, Resistance exercise performance, Skeletal muscle

## Abstract

Dietary nitrate (NO_3_^−^) can enhance skeletal muscle contractile function and explosive-type exercise by modulating type II muscle fibers; however, limited attention has been directed at exploring the optimal dosing guidelines and potential performance-enhancing effects of NO_3_^−^ supplementation during resistance-type exercise. The purpose of our investigation was to examine potential dose–response effects of concentrated NO_3_^−^-rich beetroot juice on neuromuscular performance during resistance exercise. Eighteen resistance-trained men were assigned in a double-blind, randomized, crossover design, to four conditions to consume beetroot juice containing: negligible NO_3_^−^ (PL); ~ 6 mmol NO_3_^−^ (BR-LOW); ~ 12 mmol NO_3_^−^ (BR-MOD); and ~ 24 mmol NO_3_^−^ (BR-HIGH). Participants completed 1 set of vertical countermovement jumps (CMJ), 2 sets × 3 repetitions of barbell back squats, and 2 sets × 3 repetitions of barbell bench press 2.5 h post-supplementation. Plasma [NO_3_^−^] increased in a dose-dependent manner (*P* < 0.01). Plasma [nitrite] ([NO_2_^−^]) increased in all BR conditions compared to PL (*P* < 0.05), such that BR-MOD vs. BR-LOW (*P* < 0.01) and BR-LOW vs. PL (*P* < 0.01), but BR-HIGH was not different compared to BR-MOD (*P* > 0.05). Performance was not different between conditions in CMJ, 50% one-repetition maximum (1RM) and 75%1RM back squats, or 50%1RM and 75%1RM bench press (*P* > 0.05). The change in plasma [NO_2_^−^] was significantly correlated with peak power (*r* = − 0.65, *P* = 0.003), mean power (*r* = − 0.52, *P* = 0.03), and mean velocity (*r* = − 0.48, *P* = 0.04) during 50%1RM back squats following BR-LOW vs. PL but not in other conditions (*P* > 0.05). This study indicates that dietary NO_3_^−^ does not impact resistance exercise performance at any of the doses assessed in the current study.

**Supplementary Information:**

The online version contains supplementary material available at 10.1007/s00421-025-05779-1.

## Introduction

Resistance exercise is foundational to training programs in numerous sports to increase strength and power (Currier et al. 2023). Power, which is the product of force and velocity, is a key determinant of performance in various sports (Cormie et al. 2010, 2011; Stone et al. 2003). Therefore, interventions that bolster contractile force and/or velocity are likely to have positive implications for muscle power and exercise performance in various settings. One such ergogenic supplement to enhance skeletal muscle power output is dietary nitrate (NO_3_^−^) supplementation (Coggan et al. 2021). Dietary NO_3_^−^ is purported to elicit physiological and performance effects by increasing nitric oxide (NO) bioavailability via the conversion of NO_3_^−^ to nitrite (NO_2_^−^) and then NO_2_^−^ to NO (Lundberg et al. 2008). While initially recognized for its potential to improve exercise economy and endurance performance (Bailey et al. 2009; Larsen et al. 2007), NO_3_^−^ supplementation has potential to elicit small positive effects (e.g., time trial and time-to-exhaustion performance) on performance in a variety of exercise settings (Senefeld et al. 2020).

Dietary NO_3_^−^ has been shown to improve physiological and performance responses at high compared to slow velocities (Bailey et al. 2015; Coggan et al. 2015) and high compared to low power outputs (Breese et al. 2013). In rodents, dietary NO_3_^−^ is more likely to elicit beneficial physiological effects in type II muscle (Ferguson et al. 2013; Hernández et al. 2012). Type II muscle fiber recruitment is greater (Krustrup et al. 2004) and skeletal muscle oxygenation and pH decline (Richardson et al. 1995) with increasing exercise intensity. Moreover, the reduction of NO_2_^−^ to NO is potentiated in environments of acidosis and hypoxia (Castello et al. 2006; Modin et al. 2001). These observations may underpin more recent data suggesting that dietary NO_3_^−^ is more effective during higher exercise intensities (Alsharif et al. 2023). However, while these data provide a theoretical basis for dietary NO_3_^−^ supplementation to enhance explosive exercise performance (Tan et al. 2022), available data on the performance-enhancing potential of dietary NO_3_^−^ on resistance exercise performance (i.e., power, and velocity) are limited and equivocal (Jurado-Castro et al. 2022; Ranchal-Sanchez et al. 2020; Rodríguez-Fernández et al. 2021; Tan et al. 2022; Tan et al. 2023a, b; Williams et al. 2020). Therefore, further research is required to provide insight on the efficacy of NO_3_^−^ supplementation for enhancing resistance-type exercise and the potential individual responses to NO_3_^−^ (Hopkins et al. 2009).

While the current evidence from meta-analyses suggests that the minimum effective dose for performance enhancement is an acute dose of > 5 mmol of NO_3_^−^ (i.e., at least 1 × 70 mL NO_3_^−^-rich beetroot juice ‘shot’) ingested 2.5 h prior to exercise, this guideline is based on meta-analytical data mainly from studies conducted in cycling and running (Senefeld et al. 2020; Silva et al. 2022). Thus, there is currently no recommendation or evidence on acute NO_3_^−^ supplementation protocols for enhancing resistance exercise performance. Moreover, the performance effects of NO_3_^−^ on resistance exercise may be obscured, in part, by interindividual variability in effective dosages (Tan et al. 2023a, b). For example, in bench press, an acute low NO_3_^−^ dose of (~ 6.5 mmol; 70 mL or 1 beetroot shot) has been reported to be effective (Williams et al. 2020) and ineffective (Ranchal-Sanchez et al. 2020) at enhancing power and velocity, while an acute and short-term moderate NO_3_^−^ dose (~ 13 mmol; 140 mL or 2 beetroot shots) had no effect on power and velocity (Tan et al. 2023a, b). Similarly, in back squats, an acute low NO_3_^−^ dose has been shown to be both effective (Jurado-Castro et al. 2022) and ineffective (Ranchal-Sanchez et al. 2020) at enhancing power and velocity, while an acute moderate NO_3_^−^ dose has been reported to increase peak and mean power during back squats by 15–22% (Rodríguez-Fernández et al. 2021) and to have no effect on power and velocity (Tan et al. 2022; Tan et al. 2023a, b). In countermovement jumps, an acute low NO_3_^−^ dose has been reported to increase jump height by ~ 6% in some studies (Jurado-Castro et al. 2022; López-Samanes et al. 2023), and to have no ergogenic effect in other studies (Cuenca et al. 2018; López-Samanes et al. 2022). Short-term moderate NO_3_^−^ doses have also been reported to have no effects on jump height, force (Jonvik et al. 2021; López-Samanes et al. 2022) or power (Cuenca et al. 2018). Interestingly, elevated dosages, such as 26 mmol of NO_3_^−^ (i.e., 280 ml or 4 beetroot shots), are safe and has been reported to improve electrically stimulated force production in humans (Whitfield et al. 2017), but no study has examined whether high NO_3_^−^ doses induce a more pronounced effect compared to low and moderate NO_3_^−^ doses on resistance exercise performance. Theoretically, higher NO_3_^−^ doses could further enhance physiological responses compared to lower doses as a greater magnitude of increase in NO bioavailability following NO_3_^−^ supplementation, as reflected by plasma [NO_2_^−^], has been correlated with enhanced cycling, knee extension and running performance (Coggan et al. 2018; Porcelli et al. 2015; Wilkerson et al. 2012). Thus, the optimal NO_3_^−^ dosage for enhancing resistance exercise remains unresolved.

The purpose of this study was to investigate the potential dose-response effect of concentrated NO_3_^−^-rich beetroot juice supplementation on plasma [NO_3_^−^] and [NO_2_^−^], and power and velocity during resistance exercise comprised of vertical countermovement jumps, barbell back squats bench, and barbell press. It was hypothesized that physiological and performance variables during resistance exercise would be enhanced following NO_3_^−^-rich beetroot juice and that the effects would occur in a dose-dependent effect compared to a NO_3_^−^-depleted beetroot juice.

## Materials and methods

### Participants

18 healthy resistance-trained men (mean ± SD: age 20 ± 1 years, body mass 80 ± 12 kg, height 1.77 ± 0.10 m) volunteered to participate in this study, located at Pepperdine University, following a power calculation based on a published report (Williams et al. 2020) using a power of 0.95 and alpha of 0.05, and based on an effect size of 0.96, which required *N* = 17. All participants were university students and were given a random identification code of anonymization. Resistance-trained was defined as individuals who consistently performed resistance exercise at least twice per week for at least two years prior to enrollment in the study. Participants were instructed to maintain their normal training regimens throughout the experiment. The exclusion criteria included individuals with contraindications to exercise, cardiometabolic disease, currently consuming dietary supplements containing caffeine, sodium bicarbonate, creatine, beta-alanine, and/or NO precursor supplements (i.e., NO_3_^−^, arginine, citrulline, antioxidants), women, and smokers. Women were excluded given that sex-differences in the physiological responses to NO_3_^−^ ingestion may exist (Wickham & Spriet 2019) and that including the current recommended controls (i.e., testing only during the early follicular phase) would have been unfeasible logistically (Baranauskas et al. 2022). Experimental protocols, risks, and benefits of participating were explained prior to participants providing written informed consent. This study was pre-registered on the Open Science Framework on 7 July 2023 (osf.io/uvf4w), was approved by the Institutional Research Ethics Committee (Protocol #23–03-2113), and conformed to the code of ethics of the Declaration of Helsinki.

### Experimental overview

**Fig. 1:**
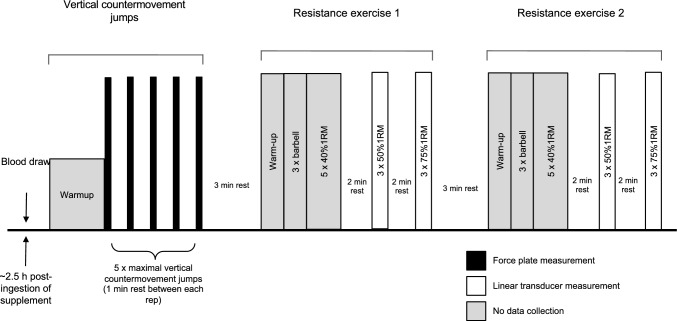
Schematic diagram of the experimental protocol

Participants reported to the laboratory on a total of six occasions over a 5-wk period **(**Fig. 1**).**

During visit 1, participants underwent standardized one-repetition maximum (1RM) testing procedures for the determination of the resistance to be applied in subsequent visits. During visit 2, participants performed a protocol and coaching technique familiarization to ensure correct lifting technique. Subsequently, in a double-blind, randomized, crossover design, participants were assigned to four experimental conditions using a web-based randomizer (random.org) to receive various acute doses of concentrated NO_3_^−^-rich beetroot juice (BR) or NO_3_^−^-depleted beetroot juice (PL), 2.5 h prior to the commencement of the exercise protocol. All supplements were identical in size, smell, taste and appearance. Each condition was separated by a wash-out period of at least 5 days given that plasma [NO_3_^−^] and [NO_2_^−^] have been shown to return to baseline 24-h post-ingestion (Wylie et al. 2013). Participants recorded their physical activity and diet during the 24 h prior to the first experimental visit (i.e., visit 3) and were asked to repeat these for subsequent visits. All tests were performed at the same time of day (± 1 h). Prior to their first visit, participants were instructed to avoid antibacterial mouthwash for the duration of the study given that mouthwash has been evidenced to interfere with NO_3_^−^ metabolism in humans (Govoni et al. 2008). Additionally, participants were required to refrain from strenuous exercise and alcohol 24 h prior to each experimental visit, and NO_3_^−^-rich foods (i.e., beetroot, celery, lettuce, radish, spinach etc.) and caffeine 12 h before each visit. The lead researcher, data collectors, and participants were blinded to the conditions. The distribution of supplements and randomization for each condition was performed by a researcher that was not formally involved in data collection processes, thereby limiting the potential of bias.

### Exercise protocols

Participants performed a warm-up in preparation for 1RM testing as previously described (Williams et al. 2020). Briefly, participants completed 5 back squats at 50% of their perceived 1RM, followed by 3 repetitions at 70% of their perceived 1RM with each set interspersed by 2 min of recovery. Subsequently, the load was increased in stepwise increments (0.2 kg to 9 kg) until the participant’s maximum was successfully lifted within 3 to 5 attempts, with each attempt interspersed by 3 min of recovery. This process was then repeated for the determination of bench press 1RM. All participants were required to use standardized procedures for the back squat (i.e., medium grip, parallel depth, neutral stance and spine, lower-body extension to original standing position), and bench press (i.e., medium grip, bar to chest, full extension of arms) throughout the entire duration of the study and were provided coaching cues to ensure standardized technique.

During visit 2, participants performed a familiarization to the exercise protocol to ensure correct jumping and lifting technique and to minimize any potential learning effects. Participants performed a standardized warm-up consisting of dynamic stretching, followed by 5 vertical countermovement jumps, interspersed by 1 min of recovery. All participants were required to use standardized procedures for the vertical countermovement jump. After 3 min of recovery, participants completed a warm-up for their randomly selected first resistance exercise (i.e., bench press or squat). Following warm-up, participants performed an explosive lift using the barbell only (20 kg) for a total of 3 repetitions. Participants then performed 1 set of 5 repetitions at 40% 1RM, 1 set of 3 repetitions at 50% 1RM, and 1 set of 3 repetitions at 75% 1RM. The same protocol was followed for the other resistance exercise following a warm-up specific to that exercise. Coaching techniques were provided during this session.

During the experimental visits (i.e., visits 3, 4, 5, 6), participants reported to the laboratory to perform the experimental protocol for the determination of primary outcomes of muscular power, velocity and explosive performance–as familiarized with on visit 2–as well as plasma [NO_3_^−^] and [NO_2_^−^] from resting venous blood samples obtained at rest 2.5 h post-supplementation in the laboratory prior to the commencement of exercise. The movement tempo of individual movement phases during resistance exercise was controlled for using an eccentric–pause–concentric–pause tempo of 1–0–1–2 to emphasize explosive movements and to standardize lifting across participants (Wilk et al. 2018). To optimize the preservation of exercise intensity, smaller muscle groups as well as fatigue-inducing exercises were performed later within the exercise protocol (e.g., unweighted low-rep ballistic exercises before loaded lifts) (American College of Sports Medicine 2009). During these visits, participants performed a 3 min standardized warm-up of dynamic stretches followed by 5 maximal vertical countermovement jumps, with each repetition interspersed by 1 min of recovery. Following this, participants had a 3 min recovery period. Then, participants completed back squats and bench press in a randomized order that was consistent within participants and across conditions, with 3 min interspersed between each exercise modality. For back squats, participants performed an unweighted cycling warm-up at 60 rpm (Monark 828E, Monark Sports and Medical, Sweden) for 3 min, and for bench press, participants performed an upper-body dynamic warm-up. Following warm-up, participants completed a second, specific warm-up consisting of 3 repetitions with the barbell only, followed by 5 repetitions at 40%1RM, with each set interspersed by 2 min of recovery. Following this, a linear position transducer (GymAware, Kinetic Performance Technology, Mitchell, Australia) was attached to the barbell to assess power and velocity of movement. Power and velocity were determined during exercise in a protocol consisting of 1 set × 3 repetitions at 50% 1RM followed by 1 set of 3 repetitions at 75% 1RM, with each set interspersed by 2 min of recovery. For both resistance exercises, participants were instructed to lift the weight as fast as possible, and encouragement and technical feedback was given to participants during all sets.

### Supplementation procedures

In a randomized, double-blinded fashion, participants were assigned to four experimental conditions to consume various combinations of NO_3_^−^-depleted beetroot juice (negligible NO_3_^−^; Beet It; James White Drinks Ltd.; Ipswich, UK) and NO_3_^−^-rich beetroot juice (~ 6.5 mmol of NO_3_^−^ per 70 ml; Beet It; James White Drinks Ltd.; Ipswich, UK): (1) four NO_3_^−^-depleted beetroot juice ‘shots’ (PL: negligible NO_3_^−^); (2) three NO_3_^−^-depleted beetroot juice ‘shots’ and one NO_3_^−^-rich beetroot juice ‘shot’ (BR-LOW: ~ 6 mmol NO_3_^−^ total); (3) two NO_3_^−^-depleted beetroot juice ‘shots’ and two NO_3_^−^-rich beetroot juice ‘shots’ (BR-MOD: ~ 12 mmol NO_3_^−^ total); and (4) four NO_3_^–^-rich beetroot juice ‘shots’ (BR-HIGH: ~ 24 mmol of NO_3_^−^). Each dosage was based on previous work in cycling exercise which demonstrated that > 8 mmol of NO_3_^−^ is required to induce physiological effects (Wylie et al. 2013). Each condition was separated by a minimum washout period of 5 days. On experimental days, participants consumed 4 × 70 ml of their allocated supplements 2.5 h before exercise given that peak plasma [NO_2_^−^] occurs ~ 2 to 3 h following NO_3_^−^ ingestion (Wylie et al. 2013). Consumption of supplements was verified via text prior to arriving at the laboratory, verbal confirmation upon arriving to the laboratory, and recorded in a log. At the start of each experimental visit, the effectiveness of the blinding procedures was assessed by verbally asking whether the participants noticed any difference in the supplements ingested.

### Measurements

#### *Plasma [NO*_*3*_^*–*^*] and [NO*_*2*_^*–*^*] analysis*

A resting venous blood sample was obtained from the antecubital vein of the forearm by a trained member of the research team upon arrival to the laboratory for the assessment of plasma [NO_3_^−^] and [NO_2_^−^]. Samples were drawn into 6 mL lithium heparin tubes (Vacutainer, Becton–Dickinson, New Jersey, USA) and centrifuged at 3100 $\times$ *g* at 4 °C for 10 min within 2 min of collection. Plasma was extracted and stored in a -80 °C freezer for the later analysis of plasma [NO_3_^−^] and [NO_2_^−^] using gas phase chemiluminescence as previously described (Tan et al. 2022). All glassware, utensils and surfaces were rinsed with deionized water to remove NO_3_^−^ and NO_2_^−^ prior to analysis. Plasma samples were thawed then deproteinized using ice-cold ethanol precipitation prior to [NO_2_^−^] analysis. Specifically, samples were centrifuged at 14,000 $\times$ *g* for 10 min, and 200 μL of the supernatant was treated with 400 μL of ice-cold ethanol. Samples were then incubated on ice for 30 min, and subsequently centrifuged at 14,000 $\times$*g* for 10 min. The [NO_2_^−^] of deproteinized plasma was determined by its reduction to NO using glacial acetic acid and aqueous sodium iodide and calibrated using sodium NO_2_^−^ standards. Following this, the deproteinized plasma samples were diluted prior to [NO_3_^−^] analysis such that 100 μL of the supernatant was added to 400 μL of deionized water. The [NO_3_^−^] of diluted deproteinized plasma was determined by its reduction to NO using vanadium chloride and hydrochloric acid and calibrated using sodium NO_3_^−^ standards. Supplements were diluted with deionized water and analyzed for [NO_3_^−^] and [NO_2_^−^] using the same methods employed for measuring plasma [NO_3_^−^] and [NO_2_^−^] and converted into mmol per 70 mL.

#### Mood

The Brunel Mood Scale (BRUMS) (Terry et al. 1999, 2003) was used to assess mood states in adult populations and was conducted prior to exercise as mood may have a mediating effect on resistance training performance (Beedie et al. 2000). Using the standard response time frame of “How do you feel right now?”, 24 items representing six subscales (i.e., anger, confusion, depression, fatigue, tension, vigor; four-items per subscale) were captured using a five-point Likert scale (i.e., 0 = not at all, 1 = a little, 2 = moderately, 3 = quite a bit, 4 = extremely). Respective items were summed so that each subscale score ranged from 0 to 16 raw points. In general, elevated vigor and decreased anger, confusion, depression, fatigue, and tension subscale scores are viewed as positive outcomes.

#### Vertical countermovement jumps

Body mass and ballistic neuromuscular performance (e.g., power, velocity, height) of the lower-body extensors were assessed during vertical countermovement jumps. Participants stood on an Advanced Mechanical Technology, Inc. (AMTI; Watertown, MA, USA) AccuPower-Optimized multi-axis force platform and were asked to jump as far upward as possible. As previously described (Petrigna et al. 2019), participants were tasked with executing a downward movement until the knees were flexed to approximately 90° and then maximally and explosively jumping upward while keeping their hands on their hips at all times. Participants were instructed to not flex their knees during the flight phase, soften their impact with their feet at landing, and give maximum explosive efforts. Following a standardized warm-up, participants performed 5 repetitions of the vertical countermovement jump with 1 min of rest between each repetition (Petrigna et al. 2019). Data were processed via AccuPower software, version 4.0 (AccuPower Solutions, Dickinson, ND, USA). During the 1 set × 5 repetitions of vertical countermovement jump, peak positive and mean concentric power, rate of power development, jump height, takeoff velocity, flight time, and peak force were recorded as was the five-repetition *average* propulsion mean force. For each repetition, the propulsion mean force represented the sum of all vertical force values divided by *N* number of data points, with *N* being the number of samples between zero velocity and take-off (Chavda et al. 2018). Per best practices, all power and force values were normalized to two-thirds body mass (Jaric et al. 2005).

#### Back squats and bench press

Power and velocity measurements were obtained during back squats and bench press using a portable, wireless, commercially available, linear position transducer (GymAware, Kinetic Performance Technology, Mitchell, Australia), which has been previously used (Tan et al. 2022; Tan et al. 2023a, b; Williams et al. 2020) and validated for test–retest reliability (Ballmann et al. 2021). During the 1 set × 3 repetitions at 50% 1RM and 1 set × 3 repetitions at 75% 1RM, power and velocity were averaged across sets for the determination of mean power and mean velocity, and the highest power and the velocity values were recorded for the determination of peak power and peak velocity. The peak and the mean power values were recorded as absolute values, as well as normalized to two-thirds body mass (Jaric et al. 2005). In our laboratory, our pilot testing using the linear transducer for back squat and bench press performance resulted in a coefficient of variation in back squats of 5% for peak power, 3% for mean power, 6% for peak velocity, and 4% for mean velocity, while the coefficient of variation in bench press was 13% for peak power, 9% mean power, and 7% for peak and mean velocity. In general, our comprehensive pilot testing results align with excellent (< 10%) to good (< 20%) coefficient of variation interpretations (Aronhime et al. 2014).

#### Force plate

All dynamic force-based metrics derived from a platform (i.e., power, jump height, force, flight time, velocity, propulsion) were obtained using an AMTI AccuPower-Optimized multi-axis portable force plate (Watertown, MA, USA) and AccuPower software version 4.0 (AccuPower Solutions, Dickinson, ND, USA) for the vertical countermovement jump at a sampling rate of 1,200 Hz. All vertical force data were left unfiltered to maintain the integrity of the raw data and because noise was not evident (Hori et al. 2009; Scott et al. 2017). AccuPower is a gold standard jumping and power analysis software. The coefficient of variation for countermovement jump height has been reported as ~ 2.5 to 5% (Cormack et al. 2008). In our laboratory, our pilot using the force plate for countermovement jump performance resulted in a coefficient of variation of 5% for jump height, 4% for power, and 7% for rate of power development.

### Statistical analyses

One-way repeated-measures ANOVAs were used to investigate statistical differences in plasma [NO_3_^−^] and [NO_2_^−^], mood, and resistance exercise performance between conditions (PL vs. BR-LOW vs. BR-MOD vs. BR-HIGH). Significant main effects were explored post hoc and pair-wise using Fisher’s least significant difference tests which do not control family-wise error rates. Rather, all pair-wise post hoc* t* tests were completed using the mean squared error (i.e., the experiment-wide error) of statistically significant ANOVAs (i.e., protected *t* tests). Pearson product-moment correlation coefficients were used to assess the relationships between changes in plasma [NO_3_^−^], [NO_2_^−^] and performance variables, in which weak, moderate, and strong correlations were operationalized as 0.2, 0.5, and 0.8, respectively. Unless stated otherwise, requisite statistical assumptions were met prior to all inferential analyses (e.g., sphericity, normality of the residuals, extreme outliers). Effect sizes for ANOVAs were measured via partial eta-squared (*η*_*p*_^2^) in which small, medium, and large effects were operationalized as 0.01, 0.06, and 0.14, respectively (Cohen 1988). Effect sizes for *t* tests were measured as hedges *g* in which small, medium, and large effects were operationalized as 0.2, 0.5, and 0.8, respectively (Cohen 1988; Lakens 2013). Statistical significance was set to *P* ≤ 0.05 with all data presented as mean ± SD, unless otherwise stated. All data were analyzed using SPSS version 27 (IBM, Armonk NY).

## Results

All eighteen participants reported consuming all servings of each supplement at the correct times and were unable to identify differences between the supplements for each condition. All participants verbally confirmed that they had maintained their habitual exercise and dietary habits, as recorded on their logs, prior to each experimental visit. All participants completed the control and treatment interventions and data from all participants were analyzed for the primary outcomes. There were reports of headaches following BR-LOW, BR-MOD, BR-HIGH (*n* = 1), nausea following BR-HIGH (*n* = 3), diarrhea following BR-MOD (*n* = 1), and brain fog following BR-MOD (*n* = 1).

### Supplement [NO_3_.^−^]

The NO_3_^−^ concentrations for PL and BR were ~ 0.05 mmol per 70 mL and ~ 5.96 mmol per 70 mL, respectively.

### Plasma [NO_3_^−^] and [NO_2_.^−^]

**Fig. 2:**
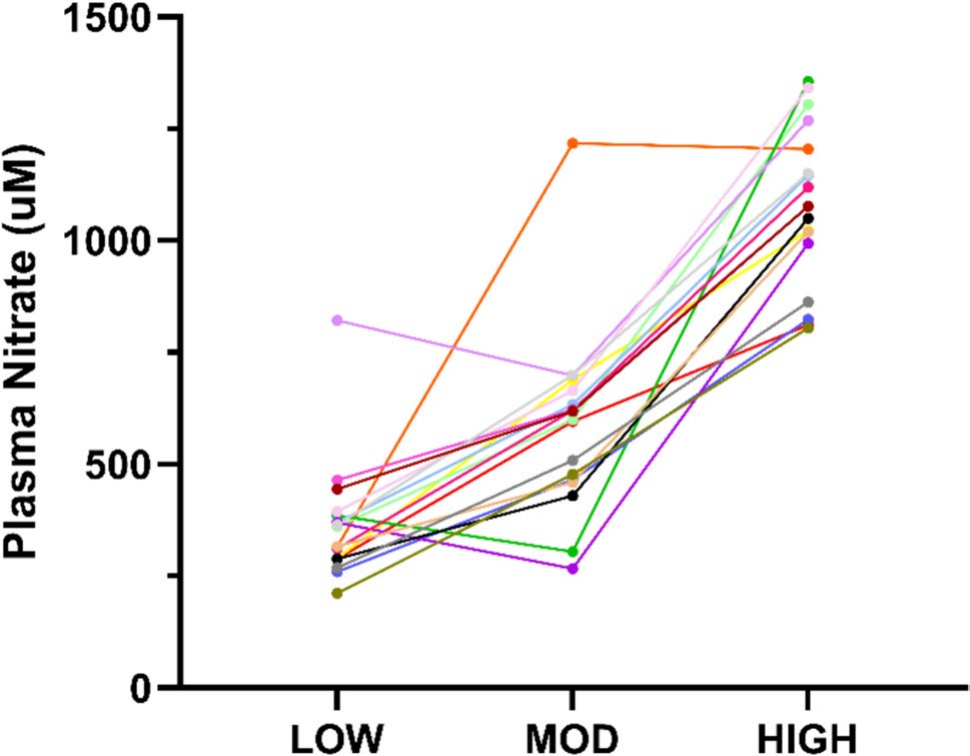
Individual plasma [nitrate] responses. HIGH = 4× nitrate-rich beetroot juice shots LOW = 1× nitrate-rich beetroot juice shot; MOD = 2× nitrate-rich beetroot juice shots

**Fig. 3:**
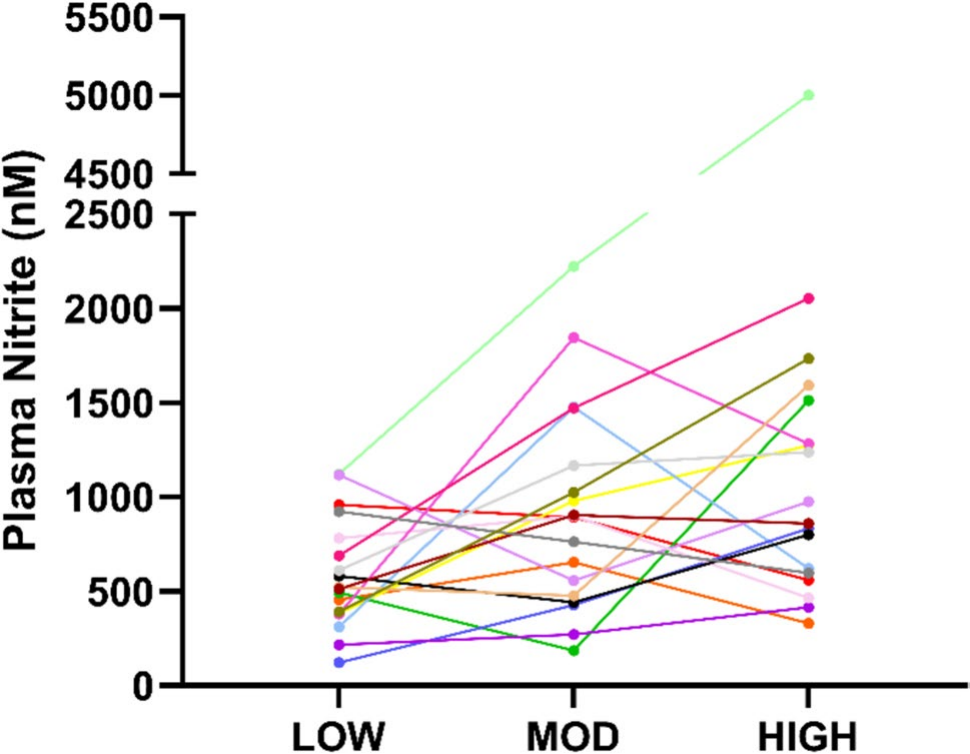
Individual plasma [nitrite] responses. HIGH = 4× nitrate-rich beetroot juice shots LOW = 1× nitrate-rich beetroot juice shot; MOD = 2× nitrate-rich beetroot juice shots

Plasma [NO_3_^−^] and [NO_2_^−^] are displayed in Table 1 and Figs. 2 and 3**.** The coefficient of variation (CV%) for duplicate samples was 1.5 ± 0.3% and 9.1 ± 10.3%, for plasma [NO_3_^−^] and [NO_2_^−^], respectively. There was a main effect by condition on plasma [NO_3_^−^] (*P* < 0.001, *n*_*p*_^2^ = 0.92) and [NO_2_^−^] (*P* < 0.001, *n*_*p*_^2^ = 0.44).

**Table 1:** Nitric oxide biomarkers following low, moderate, and high nitrate doses

Variable	PL	BR-LOW	BR-MOD	BR-HIGH
Plasma [NO_3_^−^] (µM)	39 ± 11	363 ± 132^*^	587 ± 203^*†^	1079 ± 177^*†‡^
Plasma [NO_2_^−^] (nM)	145 ± 58	587 ± 293^*^	925 ± 548^*†^	1229 ± 1063^*†^

* = significant difference compared to PL (*P* < 0.001); † = significant difference compared to BR-LOW (*P* < 0.001); ‡ = significant difference compared to BR-MOD (*P* < 0.001). BR-HIGH = 4× nitrate-rich beetroot juice shots BR-LOW = 1× nitrate-rich beetroot juice shot; BR-MOD = 2× nitrate-rich beetroot juice shots; PL = nitrate-depleted beetroot juice

Plasma [NO_3_^−^] was greater in BR-HIGH (*P* < 0.001,* g* = 5.96), BR-MOD (*P* < 0.001,* g* = 2.65), and BR-LOW (*P* < 0.001, *d*_*z*_ = 4.52) compared to PL. Plasma [NO_3_^−^] was greater in BR-HIGH (*P* < 0.001,* g* = 2.09) and BR-MOD (*P* = 0.001,* g* = 0.97) compared to BR-LOW. Plasma [NO_3_^−^] was greater in BR-HIGH (*P* < 0.001,* g* = 2.09) compared to BR-MOD.

Plasma [NO_2_^−^] was greater in BR-HIGH (*P* < 0.001,* g* = 1.99), BR-MOD (*P* < 0.001,* g* = 1.36), and BR-LOW (*P* < 0.001,* g* = 1.59) compared to PL. Plasma [NO_2_^−^] was greater in BR-HIGH (*P* < 0.001, *g* = 0.63) and BR-MOD (*P* < 0.001,* g* = 0.60) compared to BR-LOW. There was no significant difference in plasma [NO_2_^−^] between BR-HIGH and BR-MOD (*P* = 0.14).

### Correlations between plasma [NO_3_^−^] and [NO_2_^−^] and exercise performance

**Fig. 4:**
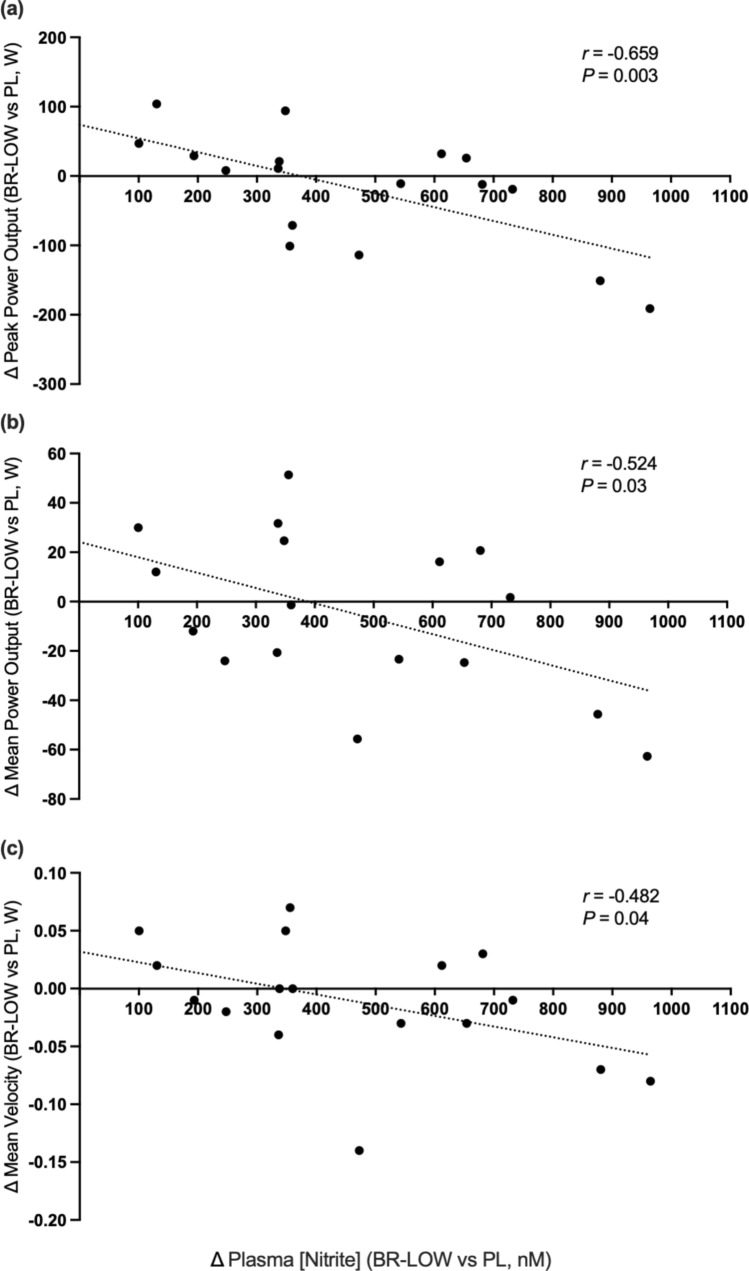
Change in plasma nitrite following BR-LOW (BR-LOW—PL) and the change in **A** peak power output, **B** mean power output, and (C) mean velocity in back squats at 50%1RM following BR-LOW (BR-LOW—PL)

No significant associations were found between the change in plasma [NO_3_^−^] and any of the performance variables. There were significant correlations between the change in plasma [NO_2_^−^] and the performance metrics during back squats at 50%1RM following BR-LOW vs PL in peak power output (*r* = − 0.65, *P* = 0.003), mean power output (*r* = − 0.52, *P* = 0.03), and mean velocity (*r* = − 0.48, *P* = 0.04) **(**Fig. 4**)**. No other significant relationships between changes in plasma [NO_2_^−^] and performance variables were uncovered.

### Mood

There was a main effect of condition on tension (*P* = 0.04, *n*_*p*_^2^ = 0.18) but no effect of condition on anger (*P* = 0.49, *n*_*p*_^2^ = 0.05), confusion (*P* = 0.25, *n*_*p*_^2^ = 0.08), depression (*P* = 0.64, *n*_*p*_^2^ = 0.03), fatigue (*P* = 0.10, *n*_*p*_^2^ = 0.12), or vigor (*P* = 0.97, *n*_*p*_^2^ = 0.00). (Table 2). However, post hoc analysis for tension was non-significant (all *P* > 0.05). Thus, the main effect finding for tension was likely due to the fact that ANOVAs account for the mean sum of squares for all of the grouping variables (i.e., ANOVAs are more likely to conclude statistical significance than targeted pair-wise tests). Based on these results, none of the mood-based variables were included as covariates within subsequent analyses.

**Table 2:** Summary of variations in mood across all experimental visits

Variable	PL		BR-LOW		BR-MOD		BR-HIGH	
	Mean	Median	Mean	Median	Mean	Median	Mean	Median
Anger	0.11 ± 0.32	0.00	0.00 ± 0.00	0.00	0.17 ± 0.70	0.00	0.00 ± 0.00	0.00
Confusion	1.00 ± 2.45	0.00	0.33 ± 0.59	0.00	0.33 ± 1.19	0.00	0.33 ± 0.77	0.00
Depression	0.06 ± 0.24	0.00	0.06 ± 0.24	0.00	0.17 ± 0.71	0.00	0.00 ± 0.00	0.00
Fatigue	2.44 ± 2.94	2.00	1.61 ± 1.58	1.50	1.67 ± 1.94	1.50	1.17 ± 1.62	1.00
Tension	0.94 ± 1.43	0.00	0.28 ± 0.96	0.00	0.28 ± 0.96	0.00	0.17 ± 0.38	0.00
Vigor	6.61 ± 3.97	6.50	6.78 ± 4.33	6.00	6.56 ± 3.89	7.00	6.61 ± 4.63	6.50

BR-HIGH = 4 × nitrate-rich beetroot juice shots; BR-LOW = 1× nitrate-rich beetroot juice shot; BR-MOD = 2× nitrate-rich beetroot juice shots; PL = nitrate-depleted beetroot juice. Data displayed as mean ± standard deviation

### Vertical countermovement jump performance

Neuromuscular performance outcomes during vertical countermovement jump are displayed in Table 3 and coefficient of variation data are in the Supplementary Materials.

**Table 3:** Vertical countermovement jump performance following low, moderate, and high nitrate doses

Variable	PL	BR-LOW	BR-MOD	BR + HIGH
Peak Force (N/Kg^0.67^)	35.91 ± 4.32	36.23 ± 5.96	35.96 ± 5.56	35.67 ± 4.93
Average Propulsion Mean Force (N/Kg^0.67^)	27.57 ± 2.54	27.50 ± 2.37	27.40 ± 2.36	27.36 ± 2.62
Rate of Power Development (W/s/Kg^0.67^)	316.57 ± 115.27	307.60 ± 105.03	306.40 ± 103.32	316.57 ± 115.27
Peak Positive Power (W/Kg^0.67^)	86.48 ± 8.13	86.42 ± 8.79	85.38 ± 8.47	84.98 ± 8.94
Concentric Mean Power (W/Kg^0.67^)	41.35 ± 6.52	41.82 ± 6.68	41.21 ± 6.65	41.36 ± 7.04
Jump Height (cm)	40.61 ± 5.62	41.15 ± 6.15	40.16 ± 5.31	40.46 ± 5.68
Takeoff Velocity (m/s)	2.82 ± 0.20	2.83 ± 0.21	2.80 ± 0.19	2.80 ± 0.20
Flight Time (s)	0.56 ± 0.04	0.57 ± 0.04	0.57 ± 0.04	0.56 ± 0.05

BR-HIGH = 4 × nitrate-rich beetroot juice shots; BR-LOW = 1 × nitrate-rich beetroot juice shot; BR-MOD = 2 × nitrate-rich beetroot juice shots; cm = centimeters; m/s = meters per second; N/Kg^0.67^ = newtons per 0.67 kg; PL = nitrate-depleted beetroot juice; W/Kg^0.67^ = watts per 0.67 kg

There was no main effect of condition on peak force (*P* = 0.63, *n*_*p*_^2^ = 0.02), average propulsion mean force (*P* = 0.73, *n*_*p*_^2^ = 0.03), rate of power development (*P* = 0.62, *n*_*p*_^2^ = 0.03), peak positive power (*P* = 0.45, *n*_*p*_^2^ = 0.05), concentric mean power (*P* = 0.63, *n*_*p*_^2^ = 0.03), jump height (*P* = 0.64, *n*_*p*_^2^ = 0.03), takeoff velocity (*P* = 0.62, *n*_*p*_^2^ = 0.03), or flight time (*P* = 0.16, *n*_*p*_^2^ = 0.10).

### Back squat performance

Neuromuscular performance outcomes during back squats at 50%1RM (141 ± 28 lbs) and 75%1RM (212 ± 42 lbs) are displayed in Table 4 and coefficient of variation data are in the Supplementary Materials.

**Table 4.** 50%1RM and 75%1RM back squat performance following low, moderate, and high nitrate doses

Variable	PL	BR-LOW	BR-MOD	BR-HIGH
50%1RM Peak Power (W)	1273.67 ± 290.48	1260.17 ± 291.73	1263.38 ± 316.12	1244.00 ± 385.21
50%1RM Peak Power (W/kg^0.67^)	23.93 ± 5.73	23.63 ± 5.26	23.56 ± 5.57	23.12 ± 6.77
50%1RM Mean Power (W)	571.76 ± 114.43	568.44 ± 123.57	565.15 ± 129.58	565.09 ± 139.28
50%1RM Mean Power (W/kg^0.67^)	10.70 ± 2.03	10.64 ± 2.07	10.52 ± 2.11	10.51 ± 2.36
50%1RM Peak Velocity (m/s)	1.52 ± 0.18	1.53 ± 0.16	1.55 ± 0.18	1.52 ± 0.21
50%1RM Mean Velocity (m/s)	0.88 ± 0.08	0.87 ± 0.09	0.87 ± 0.10	0.87 ± 0.11
75%1RM Peak Power (W)	1523.61 ± 366.83	1554.72 ± 425.24	1499.00 ± 389.65	1529.11 ± 438.56
75%1RM Peak Power (W/kg^0.67^)	28.54 ± 6.76	29.04 ± 7.01	27.95 ± 6.51	28.36 ± 7.26
75%1RM Mean Power (W)	621.89 ± 131.30	629.61 ± 148.76	617.24 ± 140.54	624.24 ± 163.37
75%1RM Mean Power (W/kg^0.67^)	11.59 ± 2.00	11.72 ± 2.22	11.48 ± 2.19	11.57 ± 2.64
75%1RM Peak Velocity (m/s)	1.30 ± 0.16	1.30 ± 0.18	1.27 ± 0.19	1.29 ± 0.21
75%1RM Mean Velocity (m/s)	0.65 ± 0.09	0.66 ± 0.10	0.65 ± 0.10	0.65 ± 0.12

BR-HIGH = 4 × nitrate-rich beetroot juice shots; BR-LOW = 1 × nitrate-rich beetroot juice shot; BR-MOD = 2 × nitrate-rich beetroot juice shots; m/s = meters per second; PL = nitrate-depleted beetroot juice; W = watts; W/kg^0.67^ = watts per 0.67 kg

There was no effect of condition on peak power (*P* = 0.78, *n*_*p*_^2^ = 0.01), mean power (*P* = 0.95, *n*_*p*_^2^ = 0.00), peak power normalized to body mass (*P* = 0.65, *n*_*p*_^2^ = 0.00), mean power normalized to body mass (*P* = 0.80, *n*_*p*_^2^ = 0.00), peak velocity (*P* = 0.78, *n*_*p*_^2^ = 0.02), mean velocity (*P* = 0.84, *n*_*p*_^2^ = 0.02) during back squats at 50%1RM. There was no effect of condition on peak power (*P* = 0.59, *n*_*p*_^2^ = 0.04), mean power (*P* = 0.83, *n*_*p*_^2^ = 0.02), peak power normalized to body mass (*P* = 0.57, *n*_*p*_^2^ = 0.01), mean power normalized to body mass (*P* = 0.74, *n*_*p*_^2^ = 0.01), peak velocity (*P* = 0.65, *n*_*p*_^2^ = 0.03), mean velocity (*P* = 0.91, *n*_*p*_^2^ = 0.01) during back squats at 75%1RM.

### Bench Press Performance

Performance outcomes during bench press at 50%1RM (110 ± 23 lbs) and 75%1RM (165 ± 35 lbs) are displayed in Table 5 and coefficient of variation data are in the Supplementary Materials.

**Table 5.** 50%1RM and 75%1RM bench press performance following low, moderate, and high nitrate doses

Variable	PL	BR-LOW	BR-MOD	BR-HIGH
50%1RM Peak Power (W)	723.22 ± 232.35	727.22 ± 252.33	720.22 ± 246.36	710.44 ± 212.44
50%1RM Peak Power (W/kg^0.67^)	13.31 ± 3.10	13.37 ± 3.56	13.24 ± 3.47	13.01 ± 2.75
50%1RM Mean Power (W)	419.24 ± 126.78	422.67 ± 131.91	422.50 ± 136.18	417.48 ± 125.87
50%1RM Mean Power (W/kg^0.67^)	7.75 ± 1.83	7.81 ± 1.93	7.80 ± 2.04	7.67 ± 7.75
50%1RM Peak Velocity (m/s)	1.21 ± 0.17	1.21 ± 0.19	1.22 ± 0.19	1.21 ± 0.20
50%1RM Mean Velocity (m/s)	0.81 ± 0.12	0.81 ± 0.12	0.81 ± 0.13	0.81 ± 0.13
75%1RM Peak Power (W)	660.83 ± 305.90	675.78 ± 277.14	635.06 ± 282.43	627.67 ± 270.18
75%1RM Peak Power (W/kg^0.67^)	12.07 ± 4.50	12.43 ± 4.22	11.63 ± 4.22	11.48 ± 3.94
75%1RM Mean Power (W)	383.61 ± 100.75	392.41 ± 114.08	384.74 ± 114.26	374.26 ± 105.69
75%1RM Mean Power (W/kg^0.67^)	7.10 ± 1.36	7.28 ± 1.65	7.09 ± 1.60	6.90 ± 1.48
75%1RM Peak Velocity (m/s)	0.77 ± 0.15	0.77 ± 0.16	0.74 ± 0.13	0.73 ± 0.15
75%1RM Mean Velocity (m/s)	0.51 ± 0.07	0.52 ± 0.11	0.51 ± 0.09	0.52 ± 0.10

BR-HIGH = 4 × nitrate-rich beetroot juice shots; BR-LOW = 1 × nitrate-rich beetroot juice shot; BR-MOD = 2 × nitrate-rich beetroot juice shots; m/s = meters per second; PL = nitrate-depleted beetroot juice; W = watts; W/kg^0.67^ = watts per 0.67 kg

There was no effect of condition on peak power (*P* = 0.82, *n*_*p*_^2^ = 0.02), mean power (*P* = 0.93, *n*_*p*_^2^ = 0.00), peak power normalized to body mass (*P* = 0.70, *n*_*p*_^2^ = 0.00), mean power normalized to body mass (*P* = 0.83, *n*_*p*_^2^ = 0.00), peak velocity (*P* = 1.00, *n*_*p*_^2^ = 0.00), mean velocity (*P* = 0.99, *n*_*p*_^2^ = 0.00) during bench press at 50%1RM. There was no effect of condition on peak power (*P* = 0.42, *n*_*p*_^2^ = 0.05), mean power (*P* = 0.34, *n*_*p*_^2^ = 0.06), peak power normalized to body mass (*P* = 0.35, *n*_*p*_^2^ = 0.09), mean power normalized to body mass (*P* = 0.24, *n*_*p*_^2^ = 0.01), peak velocity (*P* = 0.18, *n*_*p*_^2^ = 0.00), mean velocity (*P* = 0.91, *n*_*p*_^2^ = 0.00) during bench press at 75%1RM.

## Discussion

The main novel finding of this study was that there was no dose–response effect of dietary NO_3_^−^ supplementation on vertical countermovement jump, back squat and bench press performance at low, moderate, or elevated NO_3_^−^ dosages. These data are in contrast with our hypothesis and do not support acute dietary NO_3_^−^ supplementation as an ergogenic aid for enhancing resistance exercise performance in healthy resistance-trained men, at least under the conditions of this study. However, we observed significant negative correlations between the magnitude of change in plasma [NO_2_^−^] after consuming a low NO_3_^−^ dose compared to placebo and the change in 50%1RM back squat peak power, mean power, and mean velocity. These results suggest that following a low NO_3_^−^ dose (~ 6 mmol), a relatively smaller increase in plasma [NO_2_^−^] may confer more benefits to back squat performance; in contrast, relatively greater increases in plasma [NO_2_^−^] may be detrimental for back squat performance. However, we also observed that there was interindividual variability in the changes in plasma [NO_2_^−^] across all NO_3_^−^ doses. Together, our data revealed that NO_3_^−^ supplementation was ineffective at enhancing resistance exercise performance, and that the impact of variability and changes in plasma [NO_2_^−^] following NO_3_^−^ supplementation on resistance exercise performance requires further elucidation.

### Effects of dose of dietary nitrate on nitric oxide bioavailability

An original contribution of this study is that we examined if dietary NO_3_^−^ supplementation elicited dose–response effects on neuromuscular performance, for the first time, during resistance exercise, and included the highest NO_3_^−^ dose to date (24 mmol of NO_3_^−^) in a dose-response study. We observed a dose-dependent increase after acute NO_3_^−^ ingestion at dosages of ~ 6 mmol, ~ 12 mmol, and ~ 24 mmol on plasma [NO_3_^−^], which increased by ~ 9-fold, ~ 16-fold, and ~ 29-fold, respectively. Plasma [NO_2_^−^] increased by ~ 4-fold, ~ 8-fold, and ~ 10-fold, compared to PL, but there was no significant difference in plasma [NO_2_^−^] between ~ 12 mmol and ~ 24 mmol of NO_3_^−^. These results contrast an earlier NO_3_^−^ dose-response study that found dose-dependent increases at 4.2 mmol, 8.4 mmol and 16.8 mmol of NO_3_^−^ where plasma [NO_2_^−^] increased by ~ 2-fold, 4-fold, and ~ 8-fold, respectively (Wylie et al. 2013). However, our results corroborate a recent study that reported no dose-response effect above 6.4 mmol of NO_3_^−^, such that 12.8 mmol and 19.2 mmol elicited similar elevations in plasma [NO_2_^−^] (Wei et al. 2025). While the reason for this disparity is unclear, our study provided a very high dose of 24 mmol of NO_3_^−^, which may have resulted in the saturation of the oral microbiome and thus limited further increase in NO_2_^−^ production (Li et al. 1997). Therefore, our data substantiate the findings from Wei et al. and suggest that a potential saturation point for the NO_3_^−^-NO_2_^−^-NO pathway is at a dose of ~ 12 mmol of NO_3_^−^ since plasma [NO_2_^−^] did not increase with an elevated dose of 24 mmol of NO_3_^−^. Taken together, these data have important implications for NO_3_^−^ dosing strategies. However, additional research is encouraged to understand factors impacting the efficacy of NO_3_^−^ on NO bioavailability and subsequent performance effects given that to date, only three studies, including the present study, have examined NO_3_^−^ dose-response effects (Wei et al. 2025; Wylie et al. 2013).

### Effects of dose of dietary nitrate on resistance exercise performance

An original contribution of the present study was that we examined, for the first time, various dosages of NO_3_^−^ on resistance exercise performance outcomes. We found that there were no significant effects of dietary NO_3_^−^ on power and velocity metrics during vertical countermovement jumps, back squats, and bench press.

To date, our current understanding of optimal supplementation regimens for enhancing resistance exercise performance with NO_3_^−^ is limited. Of the 7 studies that have investigated the ergogenic potential of dietary NO_3_^−^ on resistance exercise, 5 studies implemented acute NO_3_^−^ ingestion 2–3 h prior to exercise (Garnacho-Castaño et al. 2022; Ranchal-Sanchez et al. 2020; Rodríguez-Fernández et al. 2021; Tan et al. 2023a, b; Williams et al. 2020) while only 2 studies implemented multiday NO_3_^−^ supplementation of 6 days (Mosher et al. 2016) and 4 days (Tan et al. 2022). In contrast, numerous studies have examined the impact of acute (2–3 h prior to exercise), short-term (3–7 days), and chronic NO_3_^−^ ingestion (≥ 7 days) on other forms of exercise (i.e., cycling and running) (Senefeld et al. 2020), highlighting the disparity in research between resistance exercise and other modalities. In cycling, time-to-exhaustion performance was improved following 8.4 mmol but not 4.2 mmol of NO_3_^−^; however, there was no further improvement between 8.4 mmol and 16.8 mmol of NO_3_^−^ (Wylie et al. 2013). In comparison, this dose-response study by Wylie et al. provided lower NO_3_^−^ doses (4.2 mmol, 8.4 mmol, 16.8 mmol) compared to our study (6 mmol, 12 mmol, 24 mmol) (Wylie et al. 2013). Thus, it is conceivable that a dose-response effect was previously observed because a lower dosing range (4.2 to 16.8 mmol) was used (Wylie et al. 2013), while in contrast, we did not observe dose-response effects because we implemented a higher dosing range (6 to 24 mmol). Taken together, these data suggest that lower doses between 4 and 8 mmol of NO_3_^−^ could have a dose-response effect on NO biomarkers and specifically for time-to-exhaustion performance in cycling (Wylie et al. 2013) but in contrast, NO_3_^−^ doses between 6 and 24 mmol of NO_3_^−^ may not have a dose-response effect on NO biomarkers or resistance exercise performance. However, further research is required to elucidate potential NO_3_^−^ dose–-esponse effects across various exercise modalities and populations because to date, only one NO_3_^−^ dose-response study has been conducted on cycling performance in males (Wylie et al. 2013), one NO_3_^−^ dose-response study has been conducted on isokinetic dynamometry in a mix of males and females (Wei et al. 2025), and our findings are the first NO_3_^−^ dose-response data for resistance exercise performance in males.

We observed significant negative correlations between the magnitude of change in plasma [NO_2_^−^] after a low NO_3_^−^ dose compared to placebo and the change in 50%1RM back squat peak power, mean power, and mean velocity. These data indicate that a low NO_3_^−^ dose (~ 6 mmol; 1 shot) concomitant with a relatively smaller increase in plasma [NO_2_^−^] improved back squat performance, while moderate (~ 12 mmol; 2 shots) and high NO_3_^−^ doses (~ 24 mmol; 4 shots) were ineffective. Notably, our results contrast earlier studies which observed that a greater increase in plasma [NO_2_^−^] following a NO_3_^−^ dose was correlated with greater performance enhancements in cycling (acute moderate NO_3_^−^ dose), knee extensions (acute moderate NO_3_^−^ dose) and running (6 days; low NO_3_^−^ dose) (Coggan et al. 2018; Porcelli et al. 2015; Wilkerson et al. 2012). However, our data and other recent advances collectively suggest the possibility that greater increases in plasma [NO_2_^−^] are not necessarily better for performance. In a study conducted in healthy older individuals, knee extension maximal power and velocity improved following a low but not a high NO_3_^−^ dose (Gallardo et al. 2021). Moreover, another study conducted in healthy adults found that knee extensor velocity improved after a low dose (~ 6.4 mmol) but not after a high NO_3_^−^ dose (~ 19.2 mmol), and that knee extensor peak and mean torque improved after a moderate dose (~ 12.8 mmol) but did not have additional enhancements following a high NO_3_^−^ dose (~ 19.2 mmol) (Wei et al. 2025). Our data extend this notion as we were surprised to see that a smaller relative increase in plasma [NO_2_^−^] was better for back squat performance following a low NO_3_^−^ dose (~ 6 mmol). While the reason for this is unclear, we speculate that differences in fiber-type recruitment patterns across exercise modalities and fiber-type composition across subject populations are important factors, especially given that NO_3_^−^ supplementation favors physiological enhancements in type II muscle fibers (Ferguson et al. 2013; Hernández et al. 2012).

The reason for the lack of overall performance effects in this study is unclear. A recent meta-analysis suggested the potential for NO_3_^−^ to have a small performance-enhancing effect on resistance exercise and highlighted that a few studies reported extremely large effects while in contrast, other studies found trivial effects (Tan et al. 2023a, b). For example, mean power (Williams et al. 2020) and peak power (Rodríguez-Fernández et al. 2021) were reported to increase by ~ 19% while others found no significant effects following NO_3_^−^ ingestion (Ranchal-Sanchez et al. 2020; Tan et al. 2022; Tan et al. 2023a, b). This heterogeneity between the limited available studies on NO_3_^−^ and resistance exercise could be due to variability in the resistance exercise protocols (e.g., intensity, modality, number of sets and repetitions, recovery time etc.). In addition, variability in the responsiveness to NO_3_^−^ may contribute to the lack of an overall ergogenic effect in resistance-type exercise but limited studies have focused on examining inter- and intra-individual variability. In the present study, we observed highly variable responses across individuals and conditions in NO biomarker and performance outcomes (Supplementary Tables 1–24**)**. For example, following 26 mmol of NO_3_^−^, mean plasma [NO_2_^−^] was 1229 nM with a range from 329 to 1591 nM. While this study did not investigate the causes of such variability, these data could suggest that an individualized dosing approach may be warranted to elicit performance enhancement effects. We speculate that the variability could be related to several factors, including differences in oral microbiota composition and activity (Vanhatalo et al. 2018), oral hygiene practices (e.g., tongue brushing) (Tribble et al. 2019) and variations in kidney excretion rates (Sundqvist et al. 2021). However, since this study, like many others in the field, was not designed to experimentally address interindividual variability or within-person response consistency, further research employing replicate-control trial designs is necessary to gain a deeper understanding of individual responses to dietary NO_3_^−^.

### Limitations and areas for further research

We were unable to include women due to the methodological, financial and logistical constraints related to the verification of hormonal profiles and testing within the early follicular phase (day 0–5) for consistency in NO levels between conditions. While imperfect, future studies may include women using calendar days and menses tracking applications to identify menstrual cycle phases or collect blood or urine samples although these methods may be more unfeasible due to cost. Importantly, while testing women within particular menstrual cycle phases is a robust study design, this approach limits external validity and “real world” application since women do not typically structure exercise regimens based on menstrual cycle phases. Areas for further research include examining if sex-differences, fiber-type composition, resistance exercise protocols, training status or other NO_3_^−^ supplementation regimens impact the efficacy of NO_3_^−^ on resistance exercise performance. Importantly, further research is required to understand how high degrees of variability impact the efficacy of dietary NO_3_^−^ for enhancing resistance exercise performance. For example, future studies may employ repeatability studies and formal statistical analysis to provide insight to interindividual variability and the minimum meaningful positive change in response to an intervention (Margaritelis et al. 2023). Lastly, future studies could include skeletal muscle [NO_3_^−^] and [NO_2_^−^], and plasma S-nitrosothiols, as these assessments may better correlate with the performance effects of NO_3_^−^.

### Conclusion

Acute dietary NO_3_^−^ supplementation provided at a low, moderate, and high dose did not impact power and velocity outcomes during explosive performance during vertical countermovement jumps or back squats and bench press in resistance-trained males. Interestingly, we found significant negative correlations between the change in 50%1RM back squat performance with the magnitude of change in plasma [NO_2_^−^] after a low NO_3_^−^ dose compared to placebo. These findings indicate that relatively greater increases in plasma [NO_2_^−^] may be detrimental to back squat performance while relatively smaller increases in plasma [NO_2_^−^] may benefit back squat performance, at least following a low NO_3_^−^ dose and under the conditions of this study. However, we observed interindividual variability in plasma [NO_2_^−^] responses across all NO_3_^−^ doses and the impact of variability on the efficacy of dietary NO_3_^−^ is currently not well-understood. Future studies are required encouraged to investigate the impact of optimal, long-term, and/or individualized NO_3_^−^ dosing approaches, as well as the impact of sex-differences, training status, interindividual variability on the efficacy of NO_3_^−^ on enhancing resistance exercise performance.

## Supplementary Information

Below is the link to the electronic supplementary material.

Supplementary file1 (DOCX 110 KB)

## Data Availability

The datasets used and/or analyzed during the current study are available from the corresponding author on reasonable request.

## Abbreviations

BR: Nitrate-rich beetroot juice
BR-LOW: 1 X nitrate-rich beetroot juice shot
BR-MOD: 2 X nitrate-rich beetroot juice shots
BR-HIGH: 4 X nitrate-rich beetroot juice shots
cm: Centimeters
m/s: Meters per second
N/Kg^0.67^: Newtons per .67 kg
NO_3_^−^: Nitrate
NO_2_^−^: Nitrite
PL: Nitrate-depleted beetroot juice
W: Watts
W/kg^0.67^: Watts per .67 kg
1RM: One-repetition maximum
