# Supplementary material for: The effect of dietary nitrate supplementation on resistance exercise performance: A dose–response investigation
Source: Eur J Appl Physiol. 2025 Apr 24;125(10):2869–83. doi: 10.1007/s00421-025-05779-1 (PMC12479694; doi:10.1007/s00421-025-05779-1)
Supplement: Supplementary file 1 — Supplementary file1 (DOCX 110 KB) [file 421_2025_5779_MOESM1_ESM.docx]

**Supplementary materials: coefficient of variation**

*Vertical Countermovement Jump Performance*

The within-day CV% for peak force, average propulsion mean force, rate of power development, peak positive power, concentric mean power, jump height, takeoff velocity and flight time were 5.4 ± 1.7%, 2.4 ± 0.6%, 11.7 ± 20.4%, 4.0 ± 2.7%, 4.7 ± 1.5%, 5.4 ± 1.5%, 2.7 ± 0.1%, and 3.3 ± 0.1%, respectively.

The between-day CV% for peak force, average propulsion mean force, rate of power development, peak positive power, concentric mean power, jump height, takeoff velocity and flight time were 3.0 ± 1.0%, 2.7 ± 0.7%, 10.5 ± 22.4%, 9.9 ± 0.6%, 6.3 ± 2.2%, 5.8 ± 1.8%, 2.9 ± 0.1%, and 2.9 ± 0.1%, respectively.

*Back Squat Performance*

The within-day CV% for peak power, mean power, peak velocity, and mean velocity during back squats at 50%1RM were 6.6 ± 54.3%, 4.6 ± 17.8%, 4.0 ± 0.1%, and 4.4 ± 0.1%, respectively. The between-day CV% for peak power, mean power, peak velocity, mean velocity during back squats at 50%1RM were 6.6 ± 54.4%, 7.1 ± 28.6%, 4.1 ± 0.01%, and 4.6 ± 0.1%, respectively.

The within-day CV% for peak power, mean power, peak velocity, and mean velocity during back squats at 75%1RM were 4.2 ± 41.0%, 4.1 ± 17.7%, 2.6 ± 0.1%, and 4.0 ± 0.1%, respectively. The between-day CV% for peak power, mean power, peak velocity, mean velocity during back squats at 75%1RM were 8.7 ± 107.7%, 4.6 ± 25.6%, 5.6 ± 0.1%, and 4.3 ± 0.1%, respectively.

*Bench Press Performance*

The within-day CV% for peak power, mean power, peak velocity, and mean velocity during bench press at 50%1RM were 6.1 ± 38.2%, 3.3 ± 11.2%, 4.5 ± 0.1%, and 3.2 ± 0.1%, respectively. The between-day CV% for peak power, mean power, peak velocity, and mean velocity during bench press at 50%1RM were 11.1 ± 56.8%, 8.5 ± 26.4%, 8.8 ± 0.1%, and 8.0 ± 0.1%, respectively.

The within-day CV% for peak power, mean power, peak velocity, and mean velocity during bench press at 75%1RM were 7.5 ± 39.4%, 1.5 ± 5.2%, 8.4 ± 0.1%, and 1.7 ± 0.1%, respectively. The between-day CV% for peak power, mean power, peak velocity, mean velocity during bench press at 75%1RM were 4.4 ± 21.8%, 3.4 ± 10.0%, 4.5 ± 0.1%, and 3.4 ± 0.1%, respectively.

**Supplementary Figure 1.**


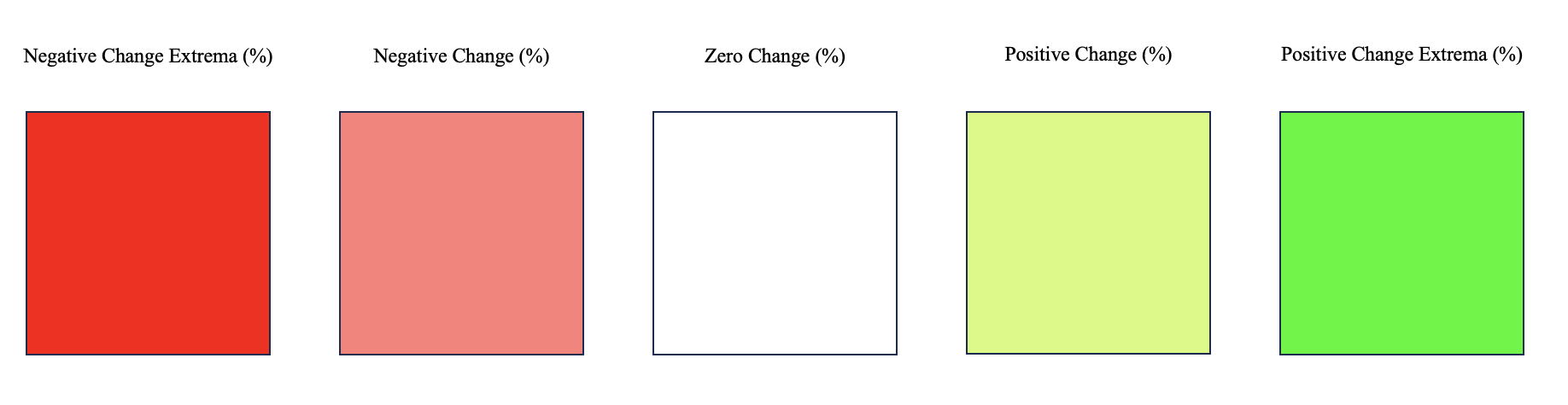


Key for descriptive information displayed in **Supplementary Tables 1-24** depicting individualized responses in % change of each individual, for each variable, across all three nitrate conditions (i.e., BR-LOW, BR-MOD, and BR-HIGH vs. PL). Specifically, % change was calculated as ([{NO_3_^-^ condition value - PL value} / PL value] * 100). Percent change values for each individual were then color-coded using one of five potential colors. Green (i.e., positive change *extrema* meaning the *largest* % change favoring a specific NO_3_^-^ condition [e.g., +4.59%]), yellow-green (i.e., positive change meaning a % change favoring a specific NO_3_^-^ condition [e.g., +1.50%]), white (i.e., no change between a NO_3_^-^ condition and PL [e.g., 0.00%]), pink (i.e., negative change meaning a % change favoring the PL condition [e.g., -1.82%]), or red (i.e., negative change *extrema* meaning the *largest* % change favoring the PL condition [e.g., -3.44%]).

| **Supplementary Table 1**. Relative change (%) in countermovement jump peak force (N/Kg^0.67^) performance. The within-day CV% for this variable was 5.4 ± 1.7%. | | | |
| --- | --- | --- | --- |
| Subject | HIGH vs PL | MOD vs PL | LOW vs PL |
| 1 | -1.83 | +4.59 | -3.17 |
| 2 | +1.50 | -2.38 | +0.23 |
| 3 | **-5.50** | -2.82 | -4.17 |
| 4 | -0.69 | +1.55 | +2.71 |
| 5 | +2.93 | -2.61 | +0.55 |
| 6 | -0.97 | +0.29 | -2.17 |
| 7 | **-7.63** | **-6.03** | -4.86 |
| 8 | +2.35 | -2.95 | -0.98 |
| 9 | +1.70 | +5.26 | +2.50 |
| 10 | +4.21 | +0.90 | **+6.73** |
| 11 | **-5.68** | -5.09 | **-8.45** |
| 12 | +2.07 | -0.67 | -1.95 |
| 13 | -5.23 | **-6.72** | -0.56 |
| 14 | -2.32 | -0.40 | +1.98 |
| 15 | +1.10 | **+18.86** | **+22.41** |
| 16 | -1.28 | +3.22 | +3.16 |
| 17 | +0.95 | **-6.99** | **-8.16** |
| 18 | -0.46 | +1.46 | **+5.73** |
| Bolded text = value exceeds within-day coefficient of variation; HIGH = 4 x 70 ml nitrate-rich beetroot juice; LOW = 1 x 70 ml nitrate-rich beetroot juice; MOD = 2 x 70 ml nitrate-rich beetroot juice; N/Kg^0.67^ = newtons per .67 kilogram; PL = nitrate-depleted beetroot juice | | | |

| **Supplementary Table 2**. Relative change (%) in countermovement jump average propulsion mean force (N/Kg^0.67^) performance. The within-day CV% for this variable was 2.4 ± 0.6%. | | | |
| --- | --- | --- | --- |
| Subject | HIGH vs PL | MOD vs PL | LOW vs PL |
| 1 | +0.24 | **+3.51** | -1.65 |
| 2 | -0.58 | +1.93 | +1.84 |
| 3 | +0.80 | **+3.43** | +0.47 |
| 4 | -1.14 | -0.06 | +0.77 |
| 5 | -1.75 | **-3.61** | **-3.15** |
| 6 | -1.89 | +0.89 | -1.42 |
| 7 | -1.56 | +1.63 | +0.17 |
| 8 | -1.82 | **-3.21** | -0.77 |
| 9 | +2.14 | **-2.93** | **+2.69** |
| 10 | -0.41 | +0.21 | +0.57 |
| 11 | **-7.78** | **-7.67** | **-7.23** |
| 12 | **+4.25** | +0.24 | -0.20 |
| 13 | **-5.94** | **-3.66** | -2.01 |
| 14 | **-4.84** | -1.24 | +0.81 |
| 15 | +1.50 | +0.29 | +1.27 |
| 16 | +0.85 | +0.13 | +0.72 |
| 17 | +2.28 | **-4.90** | **-3.73** |
| 18 | **+2.53** | **+6.08** | **+8.08** |
| Bolded text = value exceeds within-day coefficient of variation; HIGH = 4 x 70 ml nitrate-rich beetroot juice; LOW = 1 x 70 ml nitrate-rich beetroot juice; MOD = 2 x 70 ml nitrate-rich beetroot juice; N/Kg^0.67^ = newtons per .67 kilogram; PL = nitrate-depleted beetroot juice | | | |

| **Supplementary Table 3**. Relative change (%) in countermovement jump rate of power development (W/s/Kg^0.67^) performance. The within-day CV% for this variable was 11.7 ± 20.4%. | | | |
| --- | --- | --- | --- |
| Subject | HIGH vs PL | MOD vs PL | LOW vs PL |
| 1 | +4.10 | **+13.54** | -6.58 |
| 2 | +0.41 | +2.36 | +8.14 |
| 3 | +4.64 | +10.68 | +5.59 |
| 4 | -0.28 | -4.56 | +0.75 |
| 5 | +4.92 | -8.16 | -5.75 |
| 6 | +0.41 | +2.56 | -0.41 |
| 7 | -10.12 | +4.27 | -10.56 |
| 8 | +2.16 | -7.86 | +4.39 |
| 9 | **+14.69** | -10.02 | +5.96 |
| 10 | +0.18 | +2.96 | +6.27 |
| 11 | **-26.76** | **-34.87** | **-22.47** |
| 12 | **+55.75** | **-27.02** | **-29.11** |
| 13 | **-28.82** | -1.31 | **-17.29** |
| 14 | **-17.23** | -7.32 | +3.54 |
| 15 | +1.99 | -3.33 | -10.96 |
| 16 | +3.27 | +5.77 | +1.25 |
| 17 | +6.20 | **-11.90** | **-16.93** |
| 18 | -0.07 | **+17.16** | **+26.14** |
| Bolded text = value exceeds within-day coefficient of variation; HIGH = 4 x 70 ml nitrate-rich beetroot juice; LOW = 1 x 70 ml nitrate-rich beetroot juice; MOD = 2 x 70 ml nitrate-rich beetroot juice; PL = nitrate-depleted beetroot juice; W/s/Kg^0.67^ = watts per second per .67 kilogram | | | |

| **Supplementary Table 4**. Relative change (%) in countermovement jump peak positive power (W/Kg^0.67^) performance. The within-day CV% for this variable was 4.0 ± 2.7%. | | | |
| --- | --- | --- | --- |
| Subject | HIGH vs PL | MOD vs PL | LOW vs PL |
| 1 | **+5.14** | **+7.14** | **-4.76** |
| 2 | +3.69 | +1.43 | +0.92 |
| 3 | **-11.28** | **-7.34** | **-9.94** |
| 4 | -1.17 | -1.08 | -0.51 |
| 5 | +1.87 | -0.69 | **+6.03** |
| 6 | **-17.89** | **-6.25** | **-15.59** |
| 7 | -3.94 | -0.98 | -2.17 |
| 8 | -2.86 | +0.08 | -0.32 |
| 9 | +3.86 | -1.60 | **+5.68** |
| 10 | -0.70 | -2.83 | +2.89 |
| 11 | **-9.98** | -3.71 | -3.40 |
| 12 | **+11.36** | +1.05 | -2.27 |
| 13 | -2.89 | **-5.61** | **+4.87** |
| 14 | **-4.82** | -1.74 | -1.35 |
| 15 | -1.06 | -2.12 | +0.86 |
| 16 | +0.44 | +0.49 | +1.37 |
| 17 | +0.11 | -1.54 | +3.07 |
| 18 | +0.69 | +2.54 | **+16.08** |
| Bolded text = value exceeds within-day coefficient of variation; HIGH = 4 x 70 ml nitrate-rich beetroot juice; LOW = 1 x 70 ml nitrate-rich beetroot juice; MOD = 2 x 70 ml nitrate-rich beetroot juice; PL = nitrate-depleted beetroot juice; W/Kg^0.67^ = watts per .67 kilogram | | | |

| **Supplementary Table 5**. Relative change (%) in countermovement jump concentric mean power (W/Kg^0.67^) performance. The within-day CV% for this variable was 4.7 ± 1.5%. | | | |
| --- | --- | --- | --- |
| Subject | HIGH vs PL | MOD vs PL | LOW vs PL |
| 1 | +1.87 | **+7.86** | -1.17 |
| 2 | +2.89 | **+5.27** | **+6.41** |
| 3 | +2.94 | +4.42 | -0.40 |
| 4 | -1.91 | -0.39 | -0.45 |
| 5 | +0.10 | **-5.09** | -2.27 |
| 6 | -3.62 | +1.77 | -2.56 |
| 7 | -3.58 | +0.09 | -0.23 |
| 8 | -2.58 | **-5.14** | -1.46 |
| 9 | +4.69 | **-7.65** | +3.24 |
| 10 | +1.38 | -0.08 | +1.99 |
| 11 | **-10.87** | **-10.57** | -3.53 |
| 12 | **+11.68** | +2.50 | +0.08 |
| 13 | **-8.73** | -3.49 | -2.27 |
| 14 | **-7.08** | -0.99 | +0.54 |
| 15 | +2.88 | **+6.72** | **+10.90** |
| 16 | +2.76 | -2.76 | +2.00 |
| 17 | **+4.96** | -4.17 | -1.39 |
| 18 | +1.80 | **+5.88** | **+11.39** |
| Bolded text = value exceeds within-day coefficient of variation; HIGH = 4 x 70 ml nitrate-rich beetroot juice; LOW = 1 x 70 ml nitrate-rich beetroot juice; MOD = 2 x 70 ml nitrate-rich beetroot juice; PL = nitrate-depleted beetroot juice; W/Kg^0.67^ = watts per .67 kilogram | | | |

| **Supplementary Table 6**. Relative change (%) in countermovement jump height (cm) performance. The within-day CV% for this variable was 5.4 ± 1.5%. | | | |
| --- | --- | --- | --- |
| Subject | HIGH vs PL | MOD vs PL | LOW vs PL |
| 1 | **+6.90** | **+9.96** | **-6.41** |
| 2 | **+8.70** | **+6.46** | **+7.74** |
| 3 | **-13.12** | **-10.09** | **-14.18** |
| 4 | -0.93 | -1.07 | -3.08 |
| 5 | **+6.24** | -0.31 | **+14.84** |
| 6 | **-23.13** | **-11.98** | **-23.05** |
| 7 | **-6.31** | -4.97 | -1.53 |
| 8 | -3.41 | -4.60 | -1.93 |
| 9 | +4.44 | -3.57 | **+8.54** |
| 10 | +2.01 | -3.16 | +4.61 |
| 11 | **-9.25** | +4.21 | **+7.76** |
| 12 | **+18.98** | +3.45 | -1.99 |
| 13 | -1.87 | -0.92 | **+6.94** |
| 14 | +4.67 | -1.61 | -1.43 |
| 15 | +1.02 | +4.38 | **+6.05** |
| 16 | **+5.79** | -2.08 | -0.81 |
| 17 | +3.31 | +3.29 | **+10.77** |
| 18 | -3.89 | -2.79 | **+18.59** |
| Bolded text = value exceeds within-day coefficient of variation; cm = centimetres; HIGH = 4 x 70 ml nitrate-rich beetroot juice; LOW = 1 x 70 ml nitrate-rich beetroot juice; MOD = 2 x 70 ml nitrate-rich beetroot juice; PL = nitrate-depleted beetroot juice | | | |

| **Supplementary Table 7**. Relative change (%) in countermovement jump takeoff velocity (m/s) performance. The within-day CV% for this variable was 2.7 ± 0.1%. | | | |
| --- | --- | --- | --- |
| Subject | HIGH vs PL | MOD vs PL | LOW vs PL |
| 1 | **+3.09** | **+4.81** | **-3.44** |
| 2 | **+4.38** | **+3.28** | **+3.65** |
| 3 | **-6.71** | **-5.03** | **-7.38** |
| 4 | -0.34 | -0.69 | -1.72 |
| 5 | **+3.30** | +0.00 | **+7.33** |
| 6 | **-12.50** | **-6.25** | **-12.17** |
| 7 | **-3.27** | -2.55 | -0.73 |
| 8 | -1.61 | -2.25 | -0.96 |
| 9 | +2.14 | -1.78 | **+4.27** |
| 10 | +1.01 | -1.68 | +2.35 |
| 11 | **-4.53** | +2.06 | **+3.70** |
| 12 | **+9.27** | +1.93 | -0.77 |
| 13 | -0.82 | -0.41 | **+3.27** |
| 14 | -2.48 | -1.06 | -0.71 |
| 15 | +0.35 | +2.09 | **+2.79** |
| 16 | **+3.08** | -0.77 | -0.38 |
| 17 | +1.63 | +1.63 | **+5.21** |
| 18 | -1.73 | -1.38 | **+9.00** |
| Bolded text = value exceeds within-day coefficient of variation; HIGH = 4 x 70 ml nitrate-rich beetroot juice; LOW = 1 x 70 ml nitrate-rich beetroot juice; MOD = 2 x 70 ml nitrate-rich beetroot juice; m/s = metres per second; PL = nitrate-depleted beetroot juice | | | |

| **Supplementary Table 8**. Relative change (%) in countermovement jump flight time (s) performance. The within-day CV% for this variable was 3.3 ± 0.1%. | | | |
| --- | --- | --- | --- |
| Subject | HIGH vs PL | MOD vs PL | LOW vs PL |
| 1 | +0.00 | **+3.57** | +0.00 |
| 2 | +1.79 | **+3.57** | **+3.57** |
| 3 | +0.00 | +1.75 | +0.00 |
| 4 | -1.72 | +1.72 | **-3.45** |
| 5 | **+3.77** | +0.00 | +1.89 |
| 6 | -1.89 | **+11.32** | +1.89 |
| 7 | -1.72 | **-3.45** | -1.72 |
| 8 | +0.00 | +0.00 | +0.00 |
| 9 | +0.00 | **-3.51** | **-3.51** |
| 10 | +0.00 | -1.67 | +1.67 |
| 11 | **-6.12** | **+6.12** | **+4.08** |
| 12 | +0.00 | +1.92 | **-3.85** |
| 13 | **-4.08** | +0.00 | **+4.08** |
| 14 | **-5.08** | -1.69 | -1.69 |
| 15 | **+3.51** | +1.75 | **+3.51** |
| 16 | -1.89 | -1.89 | +0.00 |
| 17 | +1.56 | +3.13 | +0.00 |
| 18 | -1.72 | -1.72 | +0.00 |
| Bolded text = value exceeds within-day coefficient of variation; HIGH = 4 x 70 ml nitrate-rich beetroot juice; LOW = 1 x 70 ml nitrate-rich beetroot juice; MOD = 2 x 70 ml nitrate-rich beetroot juice; PL = nitrate-depleted beetroot juice; s = second | | | |

| **Supplementary Table 9**. Relative change (%) in 50% 1RM bench peak power (W) performance. The within-day CV% for this variable was 6.1 ± 38.2%. | | | |
| --- | --- | --- | --- |
| Subject | HIGH vs PL | MOD vs PL | LOW vs PL |
| 1 | +4.55 | +0.49 | +2.28 |
| 2 | **-18.45** | -1.79 | +2.26 |
| 3 | -0.78 | -2.19 | -2.04 |
| 4 | +1.90 | **+10.34** | +3.97 |
| 5 | +3.89 | +2.53 | **-15.56** |
| 6 | +2.48 | +5.90 | +6.10 |
| 7 | **-8.42** | -5.56 | **-9.60** |
| 8 | -1.61 | +1.17 | +3.07 |
| 9 | **+19.87** | +0.63 | +5.68 |
| 10 | -4.81 | +5.97 | **+19.88** |
| 11 | **-14.60** | -0.20 | -1.01 |
| 12 | +3.54 | +4.04 | **+6.40** |
| 13 | **+25.00** | **-9.26** | +4.44 |
| 14 | -2.95 | **-12.48** | **+9.71** |
| 15 | **-10.46** | +4.17 | **-10.37** |
| 16 | **-15.60** | -2.42 | **-15.38** |
| 17 | +3.93 | **-8.13** | -2.36 |
| 18 | -4.44 | **-7.50** | -5.21 |
| Bolded text = value exceeds within-day coefficient of variation; HIGH = 4 x 70 ml nitrate-rich beetroot juice; LOW = 1 x 70 ml nitrate-rich beetroot juice; MOD = 2 x 70 ml nitrate-rich beetroot juice; PL = nitrate-depleted beetroot juice; W = watts | | | |

| **Supplementary Table 10**. Relative change (%) in 50% 1RM bench mean power (W) performance. The within-day CV% for this variable was 3.3 ± 11.2%. | | | |
| --- | --- | --- | --- |
| Subject | HIGH vs PL | MOD vs PL | LOW vs PL |
| 1 | +2.93 | +0.26 | **-3.45** |
| 2 | **-13.61** | -1.65 | **+8.18** |
| 3 | -2.65 | **-4.70** | **-5.31** |
| 4 | **-4.17** | +1.99 | **+3.70** |
| 5 | +2.36 | **+12.16** | **-15.47** |
| 6 | **+5.35** | **+10.71** | **+10.65** |
| 7 | -3.28 | **-10.60** | **-9.83** |
| 8 | -0.16 | +0.63 | -2.04 |
| 9 | **+19.95** | **+9.62** | **+10.77** |
| 10 | **-10.02** | **+4.47** | **+3.99** |
| 11 | **-16.59** | **-4.38** | **-3.55** |
| 12 | **+7.21** | **+6.72** | **+8.47** |
| 13 | **+31.44** | **+4.99** | **+34.14** |
| 14 | **-10.04** | **-10.86** | +0.91 |
| 15 | **+12.51** | **+12.46** | -1.34 |
| 16 | **-8.84** | **-4.23** | **-11.96** |
| 17 | +0.31 | **-6.83** | **-3.53** |
| 18 | **-9.85** | **-8.55** | **-5.54** |
| Bolded text = value exceeds within-day coefficient of variation; HIGH = 4 x 70 ml nitrate-rich beetroot juice; LOW = 1 x 70 ml nitrate-rich beetroot juice; MOD = 2 x 70 ml nitrate-rich beetroot juice; PL = nitrate-depleted beetroot juice; W = watts | | | |

| **Supplementary Table 11**. Relative change (%) in 50% 1RM bench peak velocity (m/s) performance. The within-day CV% for this variable was 4.5 ± 0.1%. | | | |
| --- | --- | --- | --- |
| Subject | HIGH vs PL | MOD vs PL | LOW vs PL |
| 1 | **+8.57** | +1.90 | -1.90 |
| 2 | **-15.67** | -3.73 | +2.99 |
| 3 | -2.02 | -4.04 | -4.04 |
| 4 | +1.65 | **+9.92** | **+8.26** |
| 5 | +0.86 | +3.45 | **-14.66** |
| 6 | +2.04 | **+5.44** | +4.08 |
| 7 | -3.85 | **-7.05** | **-5.13** |
| 8 | -2.36 | +0.00 | +0.79 |
| 9 | **+14.14** | +0.00 | **+6.06** |
| 10 | **-5.65** | +3.23 | +0.00 |
| 11 | **-13.89** | -1.85 | -2.78 |
| 12 | +3.85 | +3.85 | **+4.81** |
| 13 | **+24.17** | +0.00 | **+14.17** |
| 14 | -3.85 | **-10.58** | **+4.81** |
| 15 | **+8.39** | **+9.79** | +0.70 |
| 16 | **-10.71** | -1.79 | **-13.39** |
| 17 | +1.53 | -3.05 | +0.76 |
| 18 | -2.99 | -4.48 | **-5.22** |
| Bolded text = value exceeds within-day coefficient of variation; HIGH = 4 x 70 ml nitrate-rich beetroot juice; LOW = 1 x 70 ml nitrate-rich beetroot juice; MOD = 2 x 70 ml nitrate-rich beetroot juice; m/s= metres per second; PL = nitrate-depleted beetroot juice | | | |

| **Supplementary Table 12**. Relative change (%) in 50% 1RM bench mean velocity (m/s) performance. The within-day CV% for this variable was 3.2 ± 0.1%. | | | |
| --- | --- | --- | --- |
| Subject | HIGH vs PL | MOD vs PL | LOW vs PL |
| 1 | +3.10 | +0.44 | **-3.98** |
| 2 | **-10.81** | -0.77 | **+5.79** |
| 3 | -2.78 | **-4.63** | **-5.56** |
| 4 | -2.38 | +2.78 | **+5.16** |
| 5 | +1.83 | **+11.93** | **-15.60** |
| 6 | **+3.83** | **+5.57** | **+5.57** |
| 7 | **-4.78** | **-9.22** | **-8.19** |
| 8 | -0.38 | +1.15 | -1.92 |
| 9 | **+19.79** | **+9.37** | **+10.94** |
| 10 | **-8.27** | +2.16 | +2.52 |
| 11 | **-16.67** | **-3.43** | -2.45 |
| 12 | **+7.11** | **+6.60** | **+8.12** |
| 13 | **+30.20** | **+4.46** | **+30.69** |
| 14 | **-10.05** | **-11.42** | +0.91 |
| 15 | **+5.75** | **+7.03** | **-3.51** |
| 16 | **-9.29** | **-4.87** | **-12.39** |
| 17 | +1.58 | **-3.95** | -1.58 |
| 18 | **-7.78** | **-6.67** | **-3.70** |
| Bolded text = value exceeds within-day coefficient of variation; HIGH = 4 x 70 ml nitrate-rich beetroot juice; LOW = 1 x 70 ml nitrate-rich beetroot juice; MOD = 2 x 70 ml nitrate-rich beetroot juice; m/s = metres per second; PL = nitrate-depleted beetroot juice | | | |

| **Supplementary Table 13**. Relative change (%) in 75% 1RM bench peak power (W) performance. The within-day CV% for this variable was 7.5 ± 39.4%. | | | |
| --- | --- | --- | --- |
| Subject | HIGH vs PL | MOD vs PL | LOW vs PL |
| 1 | **-24.81** | +5.19 | +2.78 |
| 2 | **-25.77** | **-9.10** | **+15.41** |
| 3 | -5.84 | **+27.04** | **-16.93** |
| 4 | **-11.00** | **-18.56** | **+40.03** |
| 5 | -3.56 | **-10.93** | **-12.35** |
| 6 | **+45.66** | **+9.31** | **+21.09** |
| 7 | -4.75 | **-10.09** | -5.82 |
| 8 | -5.13 | +0.55 | -1.83 |
| 9 | **-13.19** | **-19.37** | -4.84 |
| 10 | **-32.84** | **-7.68** | **-16.18** |
| 11 | **-9.98** | **-10.19** | **-12.06** |
| 12 | +4.29 | +5.07 | -1.75 |
| 13 | -6.54 | **-23.83** | **-9.35** |
| 14 | -3.63 | +5.98 | **+19.23** |
| 15 | **+28.44** | +0.23 | **+13.20** |
| 16 | +4.95 | **+13.80** | **+9.90** |
| 17 | +1.55 | -5.70 | **+30.22** |
| 18 | +3.38 | -2.78 | **-7.75** |
| Bolded text = value exceeds within-day coefficient of variation; HIGH = 4 x 70 ml nitrate-rich beetroot juice; LOW = 1 x 70 ml nitrate-rich beetroot juice; MOD = 2 x 70 ml nitrate-rich beetroot juice; PL = nitrate-depleted beetroot juice; W = watts | | | |

| **Supplementary Table 14**. Relative change (%) in 75% 1RM bench mean power (W) performance. The within-day CV% for this variable was 1.5 ± 5.2%. | | | |
| --- | --- | --- | --- |
| Subject | HIGH vs PL | MOD vs PL | LOW vs PL |
| 1 | **-32.49** | -1.36 | **-2.97** |
| 2 | **-6.62** | **+5.99** | **+14.60** |
| 3 | +1.45 | **+4.82** | **-14.56** |
| 4 | +0.78 | **-5.45** | **+9.24** |
| 5 | **-8.87** | **-11.67** | **+19.37** |
| 6 | **+7.89** | **+8.03** | **+9.92** |
| 7 | **-3.18** | **+1.87** | +0.44 |
| 8 | **-7.03** | **-2.37** | **-6.33** |
| 9 | **-10.97** | **-21.79** | **-20.71** |
| 10 | **-12.79** | **-1.87** | **+3.00** |
| 11 | **-3.55** | **-7.98** | **-5.58** |
| 12 | **+4.59** | **+10.55** | **+4.69** |
| 13 | **+18.25** | -0.32 | **+7.28** |
| 14 | **-2.86** | +1.43 | **+10.00** |
| 15 | **+16.42** | **+15.38** | **+13.56** |
| 16 | -1.37 | **-2.98** | -0.87 |
| 17 | **+4.46** | **+1.72** | **-4.05** |
| 18 | **-4.55** | **+1.88** | **+1.55** |
| Bolded text = value exceeds within-day coefficient of variation; HIGH = 4 x 70 ml nitrate-rich beetroot juice; LOW = 1 x 70 ml nitrate-rich beetroot juice; MOD = 2 x 70 ml nitrate-rich beetroot juice; PL = nitrate-depleted beetroot juice; W = watts | | | |

| **Supplementary Table 15**. Relative change (%) in 75% 1RM bench peak velocity (m/s) performance. The within-day CV% for this variable was 8.4 ± 0.1%. | | | |
| --- | --- | --- | --- |
| Subject | HIGH vs PL | MOD vs PL | LOW vs PL |
| 1 | +4.62 | +0.00 | -1.54 |
| 2 | **-18.99** | -5.06 | **+13.92** |
| 3 | -1.92 | **+17.31** | **-15.38** |
| 4 | -6.98 | **-15.12** | +4.65 |
| 5 | -1.45 | **-8.70** | **-14.49** |
| 6 | +2.30 | +6.90 | **+14.94** |
| 7 | -3.81 | **-11.43** | -7.62 |
| 8 | -5.41 | +0.00 | -2.70 |
| 9 | **-13.85** | **-20.00** | -6.15 |
| 10 | **-21.05** | -4.21 | **-8.42** |
| 11 | -4.29 | -7.14 | -4.29 |
| 12 | +3.17 | +6.35 | -3.17 |
| 13 | +0.00 | **-19.23** | -3.85 |
| 14 | -4.69 | -1.56 | **+15.63** |
| 15 | +6.52 | **+9.78** | +2.17 |
| 16 | -2.90 | +7.25 | -2.90 |
| 17 | +5.71 | -2.86 | **+21.43** |
| 18 | **-16.44** | -2.74 | -4.11 |
| Bolded text = value exceeds within-day coefficient of variation; HIGH = 4 x 70 ml nitrate-rich beetroot juice; LOW = 1 x 70 ml nitrate-rich beetroot juice; MOD = 2 x 70 ml nitrate-rich beetroot juice; m/s^-^= metres per second; PL = nitrate-depleted beetroot juice | | | |

| **Supplementary Table 16**. Relative change (%) in 75% 1RM bench mean velocity (m/s) performance. The within-day CV% for this variable was 1.7 ± 0.1%. | | | |
| --- | --- | --- | --- |
| Subject | HIGH vs PL | MOD vs PL | LOW vs PL |
| 1 | +1.30 | **-1.95** | **-3.25** |
| 2 | **-7.14** | **+5.84** | **+14.29** |
| 3 | +0.88 | **+4.39** | **-14.04** |
| 4 | +1.19 | **-5.36** | **+8.33** |
| 5 | **-8.78** | **-10.81** | **-13.51** |
| 6 | **+7.43** | **+8.00** | **+9.71** |
| 7 | **-3.83** | +1.64 | +0.00 |
| 8 | **+4.91** | **-3.07** | **-6.75** |
| 9 | **-11.49** | **-22.30** | **-20.95** |
| 10 | **-12.72** | **-2.31** | **+2.89** |
| 11 | **-2.34** | **-7.03** | **-4.69** |
| 12 | **+4.55** | **+9.85** | **+3.79** |
| 13 | **+19.08** | +0.00 | **+8.09** |
| 14 | **-2.16** | **+2.16** | **+10.07** |
| 15 | **+14.97** | **+13.71** | **+13.71** |
| 16 | -1.33 | **-2.67** | -0.67 |
| 17 | **+4.44** | +1.48 | **-5.19** |
| 18 | **-4.23** | **+2.82** | +1.41 |
| Bolded text = value exceeds within-day coefficient of variation; HIGH = 4 x 70 ml nitrate-rich beetroot juice; LOW = 1 x 70 ml nitrate-rich beetroot juice; MOD = 2 x 70 ml nitrate-rich beetroot juice; m/s = metres per second; PL = nitrate-depleted beetroot juice | | | |

| **Supplementary Table 17**. Relative change (%) in 50% 1RM squat peak power (W) performance. The within-day CV% for this variable was 6.6 ± 54.3%. | | | |
| --- | --- | --- | --- |
| Subject | HIGH vs PL | MOD vs PL | LOW vs PL |
| 1 | -5.44 | -2.68 | -0.97 |
| 2 | **-16.60** | -4.78 | -6.03 |
| 3 | **-7.88** | **-6.79** | +2.12 |
| 4 | **-11.13** | **-10.12** | **-14.76** |
| 5 | **+25.20** | **+9.27** | **+12.27** |
| 6 | **+14.52** | **+25.97** | **+9.38** |
| 7 | **+18.42** | **+10.46** | +4.42 |
| 8 | **-6.69** | -3.06 | **-9.62** |
| 9 | **-12.93** | **-7.76** | +3.57 |
| 10 | +4.65 | **+8.20** | +1.51 |
| 11 | -1.27 | +3.64 | +1.00 |
| 12 | **-33.69** | **-23.24** | +2.42 |
| 13 | **-22.05** | **-8.04** | **-14.79** |
| 14 | **-20.69** | -1.08 | -0.85 |
| 15 | **+11.49** | **+8.52** | +1.42 |
| 16 | **-13.58** | **-11.68** | **-9.01** |
| 17 | **+34.36** | -1.60 | -1.17 |
| 18 | -3.84 | +0.40 | +0.64 |
| Bolded text = value exceeds within-day coefficient of variation; HIGH = 4 x 70 ml nitrate-rich beetroot juice; LOW = 1 x 70 ml nitrate-rich beetroot juice; MOD = 2 x 70 ml nitrate-rich beetroot juice; PL = nitrate-depleted beetroot juice; W = watts | | | |

| **Supplementary Table 18**. Relative change (%) in 50% 1RM squat mean power (W) performance. The within-day CV% for this variable was 4.6 ± 17.8%. | | | |
| --- | --- | --- | --- |
| Subject | HIGH vs PL | MOD vs PL | LOW vs PL |
| 1 | **-6.56** | -0.79 | +3.25 |
| 2 | **-4.97** | **+10.85** | **+8.32** |
| 3 | -2.71 | +0.83 | -1.88 |
| 4 | **-6.71** | **-7.09** | **-11.68** |
| 5 | **+14.02** | **+5.50** | **+5.90** |
| 6 | +2.31 | **+16.55** | +2.25 |
| 7 | **+9.17** | **+5.38** | +3.62 |
| 8 | -1.01 | **-6.47** | **-7.27** |
| 9 | -4.13 | **-9.15** | **+5.38** |
| 10 | +3.66 | **+4.61** | -3.05 |
| 11 | -2.70 | -0.61 | -4.18 |
| 12 | **-21.96** | **-23.45** | +2.57 |
| 13 | **-7.06** | -4.52 | **-15.11** |
| 14 | **-11.45** | -1.83 | -3.89 |
| 15 | **+5.37** | +3.10 | **+4.90** |
| 16 | **-5.12** | **-7.73** | -0.39 |
| 17 | **+23.29** | -1.64 | +0.24 |
| 18 | **-10.04** | **-7.86** | -4.49 |
| Bolded text = value exceeds within-day coefficient of variation; HIGH = 4 x 70 ml nitrate-rich beetroot juice; LOW = 1 x 70 ml nitrate-rich beetroot juice; MOD = 2 x 70 ml nitrate-rich beetroot juice; PL = nitrate-depleted beetroot juice; W = watts | | | |

| **Supplementary Table 19**. Relative change (%) in 50% 1RM squat peak velocity (m/s) performance. The within-day CV% for this variable was 4.0 ± 0.1%. | | | |
| --- | --- | --- | --- |
| Subject | HIGH vs PL | MOD vs PL | LOW vs PL |
| 1 | **+26.27** | **+28.81** | **+32.20** |
| 2 | **-8.88** | +0.59 | -3.55 |
| 3 | **-4.48** | -3.73 | -1.49 |
| 4 | -3.59 | **-5.99** | **-8.38** |
| 5 | **+24.00** | **+10.40** | **+10.40** |
| 6 | **+5.16** | **+12.90** | +3.87 |
| 7 | **+4.85** | **+6.06** | **+5.45** |
| 8 | -1.70 | -1.70 | **-7.39** |
| 9 | **-12.03** | **-8.23** | -1.90 |
| 10 | +3.29 | +3.29 | +0.00 |
| 11 | -0.71 | +0.71 | +0.00 |
| 12 | **-6.72** | +0.00 | +3.73 |
| 13 | **-13.51** | -2.70 | **-13.51** |
| 14 | **-13.33** | -2.00 | -2.00 |
| 15 | **+6.63** | **+5.52** | +3.31 |
| 16 | **-7.19** | **-7.19** | **-5.04** |
| 17 | **+17.09** | +1.27 | +2.53 |
| 18 | **-6.36** | **-5.20** | -0.58 |
| Bolded text = value exceeds within-day coefficient of variation; HIGH = 4 x 70 ml nitrate-rich beetroot juice; LOW = 1 x 70 ml nitrate-rich beetroot juice; MOD = 2 x 70 ml nitrate-rich beetroot juice; m/s = metres per second; PL = nitrate-depleted beetroot juice | | | |

| **Supplementary Table 20**. Relative change (%) in 50% 1RM squat mean velocity (m/s) performance. The within-day CV% for this variable was 4.4 ± 0.1%. | | | |
| --- | --- | --- | --- |
| Subject | HIGH vs PL | MOD vs PL | LOW vs PL |
| 1 | **-6.64** | -1.05 | +3.15 |
| 2 | -2.43 | **+10.12** | **+8.10** |
| 3 | -2.23 | +0.89 | -1.79 |
| 4 | **-4.98** | -4.27 | **-8.90** |
| 5 | **+13.75** | **+5.83** | **+5.83** |
| 6 | +2.23 | **+10.97** | +1.86 |
| 7 | **+5.61** | **+5.28** | +2.64 |
| 8 | -1.42 | **-5.69** | **-7.83** |
| 9 | -4.07 | **-8.94** | **+5.69** |
| 10 | +2.26 | +3.38 | -3.01 |
| 11 | -2.56 | -0.43 | **-4.70** |
| 12 | +0.00 | -2.16 | +2.80 |
| 13 | **-6.72** | **-4.85** | **-15.30** |
| 14 | **-11.67** | -1.95 | -3.89 |
| 15 | +2.55 | +0.32 | +0.00 |
| 16 | **-5.44** | **-7.95** | -0.42 |
| 17 | **+13.06** | -1.87 | -0.75 |
| 18 | **-7.58** | **-5.05** | -2.53 |
| Bolded text = value exceeds within-day coefficient of variation; HIGH = 4 x 70 ml nitrate-rich beetroot juice; LOW = 1 x 70 ml nitrate-rich beetroot juice; MOD = 2 x 70 ml nitrate-rich beetroot juice; m/s = metres per second; PL = nitrate-depleted beetroot juice | | | |

| **Supplementary Table 21**. Relative change (%) in 75% 1RM squat peak power (W) performance. The within-day CV% for this variable was 4.2 ± 41.0%. | | | |
| --- | --- | --- | --- |
| Subject | HIGH vs PL | MOD vs PL | LOW vs PL |
| 1 | **+5.53** | +3.37 | **+12.42** |
| 2 | **+4.34** | **+7.13** | **+8.31** |
| 3 | +3.75 | **-10.96** | **+21.85** |
| 4 | **-13.50** | **-13.56** | **-13.62** |
| 5 | **+13.67** | **+8.47** | -2.05 |
| 6 | +3.91 | +0.75 | **-10.59** |
| 7 | **+4.99** | -1.08 | -1.14 |
| 8 | **-14.16** | **-16.82** | **-6.19** |
| 9 | **-12.93** | **-19.62** | **-16.80** |
| 10 | +1.47 | **+4.28** | **+11.29** |
| 11 | -1.84 | **-5.24** | -3.68 |
| 12 | **-11.66** | -3.86 | **+4.94** |
| 13 | **-6.92** | +3.23 | **+6.00** |
| 14 | **-17.76** | +0.26 | **-15.32** |
| 15 | **+29.27** | **+19.09** | **+30.48** |
| 16 | **-11.76** | +0.11 | **+6.38** |
| 17 | **+17.94** | -1.06 | +0.11 |
| 18 | +3.80 | -0.06 | **+7.85** |
| Bolded text = value exceeds within-day coefficient of variation; HIGH = 4 x 70 ml nitrate-rich beetroot juice; LOW = 1 x 70 ml nitrate-rich beetroot juice; MOD = 2 x 70 ml nitrate-rich beetroot juice; PL = nitrate-depleted beetroot juice; W = watts | | | |

| **Supplementary Table 22**. Relative change (%) in 75% 1RM squat mean power (W) performance. The within-day CV% for this variable was 4.1 ± 17.7%. | | | |
| --- | --- | --- | --- |
| Subject | HIGH vs PL | MOD vs PL | LOW vs PL |
| 1 | -3.70 | -1.36 | **+12.38** |
| 2 | -1.39 | **+12.40** | **+9.89** |
| 3 | **+8.90** | +0.49 | **+13.06** |
| 4 | **-9.39** | **-5.37** | **-12.51** |
| 5 | **+5.56** | -2.84 | **-7.05** |
| 6 | **+7.59** | **+7.38** | +3.69 |
| 7 | -0.58 | **-9.55** | -3.44 |
| 8 | +3.58 | -2.13 | -0.05 |
| 9 | **+6.06** | **-6.81** | -0.63 |
| 10 | +0.29 | -0.82 | -1.36 |
| 11 | **-5.00** | **-11.24** | **-13.54** |
| 12 | **-33.21** | -3.03 | -0.97 |
| 13 | +3.13 | +0.88 | -0.56 |
| 14 | **-9.96** | **-6.09** | -3.10 |
| 15 | **+7.89** | **+10.51** | **+10.95** |
| 16 | **-5.28** | **-4.66** | -0.81 |
| 17 | **+19.77** | +0.17 | +3.38 |
| 18 | +2.49 | **+5.90** | **+7.60** |
| Bolded text = value exceeds within-day coefficient of variation; HIGH = 4 x 70 ml nitrate-rich beetroot juice; LOW = 1 x 70 ml nitrate-rich beetroot juice; MOD = 2 x 70 ml nitrate-rich beetroot juice; PL = nitrate-depleted beetroot juice; W = watts | | | |

| **Supplementary Table 23**. Relative change (%) in 75% 1RM squat peak velocity (m/s) performance. The within-day CV% for this variable was 2.6 ± 0.1%. | | | |
| --- | --- | --- | --- |
| Subject | HIGH vs PL | MOD vs PL | LOW vs PL |
| 1 | **+3.39** | +1.69 | **+7.63** |
| 2 | **-4.80** | +0.00 | **+7.20** |
| 3 | **+5.38** | **-6.45** | **+17.20** |
| 4 | **-13.16** | **-9.87** | **-12.50** |
| 5 | **+6.72** | **+5.88** | **-4.20** |
| 6 | **+5.56** | **+5.56** | **-3.47** |
| 7 | **+3.38** | **-5.41** | +0.00 |
| 8 | **-7.84** | **-12.42** | **-6.54** |
| 9 | **-7.87** | **-17.32** | **-17.32** |
| 10 | **+3.65** | +0.73 | **+7.30** |
| 11 | +0.83 | **-5.79** | **-5.79** |
| 12 | **-7.83** | **-4.35** | -0.87 |
| 13 | **-6.45** | **-3.23** | +2.42 |
| 14 | **-15.32** | -2.42 | **-10.48** |
| 15 | **+17.69** | **+14.97** | **+17.69** |
| 16 | **-9.65** | -0.88 | **+5.26** |
| 17 | **+15.38** | +1.54 | +2.31 |
| 18 | +1.39 | +1.39 | **+4.17** |
| Bolded text = value exceeds within-day coefficient of variation; HIGH = 4 x 70 ml nitrate-rich beetroot juice; LOW = 1 x 70 ml nitrate-rich beetroot juice; MOD = 2 x 70 ml nitrate-rich beetroot juice; m/s = metres per second; PL = nitrate-depleted beetroot juice | | | |

| **Supplementary Table 24**. Relative change (%) in 75% 1RM squat mean velocity (m/s) performance. The within-day CV% for this variable was 4.0 ± 0.1%. | | | |
| --- | --- | --- | --- |
| Subject | HIGH vs PL | MOD vs PL | LOW vs PL |
| 1 | -3.30 | -0.94 | **+12.74** |
| 2 | +0.00 | **+12.20** | **+7.93** |
| 3 | **+8.97** | +0.69 | **+13.10** |
| 4 | **-6.79** | -2.71 | **-11.31** |
| 5 | **+6.06** | -3.03 | **-7.07** |
| 6 | **+7.41** | **+5.56** | +2.31 |
| 7 | -1.61 | **-9.27** | **-4.03** |
| 8 | +3.52 | -2.51 | +0.00 |
| 9 | **+6.43** | **-7.02** | -0.58 |
| 10 | -2.90 | -2.90 | **-4.35** |
| 11 | **-5.00** | **-11.67** | **-13.33** |
| 12 | **-32.76** | -2.87 | +0.00 |
| 13 | +3.45 | +1.48 | +0.00 |
| 14 | **-9.66** | **-5.68** | -3.41 |
| 15 | **+4.84** | **+7.66** | **+6.05** |
| 16 | **-4.09** | -3.51 | +0.00 |
| 17 | **+13.93** | +0.50 | +2.49 |
| 18 | +3.06 | **+7.65** | **+8.67** |
| Bolded text = value exceeds within-day coefficient of variation; HIGH = 4 x 70 ml nitrate-rich beetroot juice; LOW = 1 x 70 ml nitrate-rich beetroot juice; MOD = 2 x 70 ml nitrate-rich beetroot juice; m/s = metres per second; PL = nitrate-depleted beetroot juice | | | |
